# Supplementary material for: Micronutrients and Major Depression: A Mendelian Randomisation Study
Source: Nutrients. 2024 Oct 29;16(21):3690. doi: 10.3390/nu16213690 (PMC11547740; doi:10.3390/nu16213690)
Supplement: Supplementary file 1 [file nutrients-16-03690-s001.zip › nutrients-3207650-supplementary.pdf]

Supplementary Material

Supplementary Tables ... **2**
Supplementary Table S1: Previous MR Studies of Micronutrient Exposures in Depression ... 2
Supplementary Table S2: Characteristics of the PGC MDD GWAS ... 5
Supplementary Table S3: Studies contributing data for micronutrient GWASs with PGC MDD sample overlap ... 6
Supplementary Table S4: Micronutrient instruments used in analyses ... 12
Supplementary Table S5: Heterogeneity Statistics for MR analyses ... 19
Supplementary Table S6: Table containing SE_{IVW}/SE_{dIVW} ratios for relevant micronutrients. ... 21
Supplementary Table S7: Heterogeneity analyses for reverse MR analyses ... 22

Supplementary Material ... **23**
Supplementary Material S1: Supplementary Methods ... 23
Supplementary Material S2: Individual Micronutrient MR plots ... 24
Supplementary Material S3: List of PGC MDD collaborators ... 54

References ... **66**

Supplementary Tables

Supplementary Table S1: Previous MR Studies of Micronutrient Exposures in Depression

Exposure	Study	Outcome Sample	Exposure Sample Size	Outcome Sample Size	MR Method	SNPs	Results (OR or beta; (95% confidence intervals)[1]
**Vitamin D**	Michaelsson 2018[1]	Psychiatric Genomics Consortium (PGC)	79,366	173,005	Two-sample	6	0.98 (0.93, 1.03) p=0.44[2]
	Milaneschi 2019[2]	Netherlands Study of Depression and Anxiety (NESDA)	2,013	2,047	One-sample	6	0.94 (0.84, 1.06) p=0.34
		PGC	79,366	480,359	Two -sample	6	0.97 (0.88, 1.07)[3] p=0.50
	Mulugeta 2020[3]	PGC	79,366	331,677	Two sample	6	0.97 per 50% increase in vit D (0.91, 1.04)
		UK Biobank (UKBB)	79,366	251,962	Two sample	6	0.97 per 50% increase (0.90, 1.05)

[1] Results are given per SD change in exposure unless otherwise stated. p-values are included where in original publication
[2] Original paper gives OR in terms of decrease in vitamin D levels, i.e., OR: 1.02 per SD decrease in vitamin D levels (95% confidence intervals: 0.97, 1.08)
[3] Original paper gives OR in log odds ratio. OR converted from publication data: -0.02 (SE: 0.011) p=0.029

	Libuda 2019[4]	UKBB Depressive Symptoms	79,366	161,460	Two sample	6	0.025 (-0.05, 0.10) p=0.52
		UKBB Depression	79,366	322,580	Two-sample	6	0.98 (0.96, 1.00) p=0.10
	Revez 2020[5]	PGC	417,580	807,553	Two-sample	240	0.98 (0.96, 1.00) p=0.03[b]
**B12**	Mollehave 2017[6]	Health 2006 & Inter 99	4,126	4,126	One-sample	12	0.96 (0.52, 1.79) p=0.91
**Folate**		Health 2006 & Inter 99	3,942	3,942	One-sample	2	1.18 (0.18, 7.66) p=0.86
**Homocysteine**	Yu 2022	PGC	44,147	42,455	Two-sample	13	0.95 (0.88, 1.00) p=0.12
**Calcium**	Cheng 2019[7]	Chinese Women through CONVERGE consortium	39,400	10,640	Two-sample	6	0.92 (0.67, 1.28) p=0.63
**Magnesium**			15,366			3	1.19 (0.22, 6.61) p=0.84
**Iron**			48,972			9	0.98 (0.91, 1.05) p=0.60
**Zinc**			2,603			2	0.99 (0.95, 1.03) p=0.66

Supplementary Table S2: Characteristics of the PGC MDD GWAS

Summary statistics from the Psychiatric Genomics Consortium MDD GWAS published in 2019[8] were used as the primary outcome sample. This GWAS is a meta-analysis of the three largest independent GWASs of MDD to date, including 246,363 cases and 561,190 controls. 23andMe Participants provided informed consent and participated in the research online, under a protocol approved by the external AAHRPP-accredited IRB, Ethical & Independent Review Services (E&I Review). For full details of the methods used for genotyping in each cohort, please refer to the original publications.

Author	Sample	N	MDD categorisation
Hyde et al[9]	23andMe discovery cohort/307k	75,607 cases<br>231,747 controls	Self-report clinical diagnosis
Howard et al[8]	UK Biobank	127,552 cases<br>233,763 controls	Self-reported help seeking for problems with nerves, anxiety, tension, or depression
Wray et al[10]	Total sample (five cohorts)	43,204 cases<br>95,680 controls	See below for details. Individual cohort numbers below are prior to exclusion of 23andMe_307k and prior release of UK Biobank data (N=4,381)
	PGC 29	16,823 cases<br>25,632 controls	Structured diagnostic interviews from 29 samples as described in Wray et al.[10]
	deCODE	1,980 cases<br>9,536 controls	National inpatient electronic records
	GenScotland	997 cases<br>6,358 controls	Structured diagnostic interview
	GERA	7,162 cases<br>38,307 controls	Kaiser Permanenta Northern California Electronic Medical records (1995-2013)
	iPSYCH	18,629 cases<br>17,841 controls	National inpatient electronic records

Supplementary Table S3: Studies contributing data for micronutrient GWASs with PGC MDD sample overlap

Group	*Exposure*	*Measure*	*Author*	*Population*	*Study*	*Cohort contributes data to PGC*	*N*
Water soluble Vitamins	Vitamin B6	Serum B6	Tanaka[11]	Italy	InCHIANTI	No	1178
				Italy	Progetto Nutrizione	No	686
	Vitamin B9	Serum Folate	Grarup[12]	Iceland	Icelandic	No	20,717
				Denmark	Inter-99	No	5,624
				Denmark	Health 2006	No	2,804
		Serum Homocysteine	Van Meurs[13]	Italy	InCHIANTI	No	1,208
				USA	Baltimore Longitudinal Study of Aging	No	638
				USA/ UK	Nurse's Health Study	No	1,658
				USA	Women's Genome Health Study		13,974
				Netherlands	Rotterdam Study I	Yes	3,414
				Netherlands	Rotterdam Study II	Yes	1,868
				UK	TwinsUK cohort	No	1,172
				Switzerland	CoLaus cohort	Yes	5,434
				USA	Cardiovascular Health Study	No	3,980
				USA	Framingham Heart Study	No	10,251*
				Netherlands	Nijemen Medical Study	No	550
	Vitamin B12	Serum B12	Grarup[12]	Icelandic	Icelandic	No	25,960
				Danish	Inter-99	No	5,481
				Danish	Health 2006	No	2,812

	Vitamin C	Plasma Vitamin C	Zheng 2021	UK	Fenland	No	10,771
				UK	EPIC-Norfolk	No	16,756
				France, Italy, Spain, UK, Netherlands, Germany, Sweden, Denmark	EPIC-InterAct	No	16,841
				As above	EPIC-CVD	No	7,650
Fat soluble Vitamins	Vitamin A	Serum Retinol	Mondul[14]	Finland	Alpha-Tocopherol, Beta Carotene Cancer Prevention (ATBC) Study	No	4,014
				USA	Prostate, Lung, Colorectal and Ovarian Cancer Screening trial	No	992
				UK	Nurses Health Study (NHS)	No	2772
				Italy	InCHIANTI	No	1,124
		Beta Carotene	Ferrucci[15]	Italy	InCHIANTI	No	1,191
				USA	Women's Health and Aging Study	No	615
				USA	ATBC Study	No	2,136
	Vitamin D	Serum 25(OH)D	Jiang[16]	UK	1958 British Birth Cohort	No	4,985
				USA	The Cardiovascular Health Study	No	1,791
				USA	The Framingham Heart Study	No	5,654
				Sweden	The Gothenburg Osteoporosis and Obesity Determinants Study	No	921
				USA	The Health, Aging, Body Composition Study	No	1,558
				USA	The Indiana Women cohort	No	567
				Finland	The North Finland Birth Cohort 1966	No	4,604
				USA	The Old Order Amish Study	No	330
				Netherlands	The Rotterdam Study	Yes	1,237
				UK	Twins UK	No	5,135

	Finland	ATBC	No	1,372
	USA	PLCO	No	1,315
	USA	Atherosclerosis Risk in Communities (ARIC)	No	8,124
	Germany	AtheroGene	No	1,062
	Netherlands	B-vitamins for the Prevention of Osteoporotic Fractures (BPROOF)	No	2,525
	USA	Epidemiology of Diabetes Interventions and Complications (EDIC)	No	1,094
	Finland	Case- Control Study for Metabolic Syndrome (GENMETS)	No	1,641
	Finland	The Helsinki Birth Cohort Study (HBCS)	No	917
	USA	Coronary Heart Disease Case - Control Study nested within NHS and HPFS (HPFS_CHD)	No	1,245
	Italy	The Invecchiare in Chianti Study (InChianti)	No	1,094
	Germany	Cooperative Health Research in the Region Augsburg (KORA)	Yes (controls)	1,805
	Netherlands	Leiden Longevity Study (LLS)	No	2,265
	Germany	Ludwigshafen Risk and Cardiovascular Health Study (LURIC)	No	2,846
	USA	Multi -Ethnic Study of Atherosclerosis (MESA)	No	2,240
	Netherlands	Nijmegen Biomedische Studie	No	2,610
	UK/ USA	Breast Cancer Case-Control Study nestled in NHS	No	870
	UK/ USA	Type II diabetes Case-Control Study nested within NHS	No	724
	Scotland	The Orkney Complex Disease Study (ORCADES)	No	847
	Scotland, Ireland and the Netherlands	Prospective Study of Pravastatin in the Elderly at Risk (PROSPER)	No	4,871
	Netherlands	Rotterdam Study I& II	Yes	8,313
	Germany	The Study of Health in Pomerania (SHIP)	Yes	1,655
2	UK	The Scottish Colorectal Cancer Study	No	1,165
	Finland	Cardiovascular risk in Young Finns Study	No	1,984
	Sweden	Prospective Investigation of the Vasculature in Uppsala Seniors (PIVUS)	No	989

				Sweden	Uppsala Longitudinal Study of Adult Men (ULSAM)	No	1,124
Macrominerals	Calcium	Serum Calcium	O'Seaghadha[17]	Iceland	Age Gene/Environment Susceptibility (AGES)	No	1949
				USA	ARIC	No	9049
				USA	Baltimore Study of Aging (BLSA)	No	719
				USA	CHS	No	1802
				Switzerland	CoLaus	Yes	5411
				Croatia	CROATIA-Korcula	No	880
				Croatia	CROATIA-Split	No	488
				Croatia	CROATIA-Vis	No	910
				USA	FHS	No	2853
					HABC	No	1554
				Italy	InCHIANTI	No	1204
				UK	LBC1936	No	993
				UK	LOLIPOP EW A	No	589
				UK	LOLIPOP EW P	No	652
				UK	LOLIPOP EW610	No	927
				Sardinia	OGP Talana	No	1039
				UK	ORCADES	No	877
				Netherlands	Rotterdam Study	No	3436
				Germany	SHIP	Yes	4068
				UK	British Genetics of Hypertension Study (BRIGHT)	No	1855
				Switzerland	Bus Santé	No	4670
				Italy	INGI-Carlantino	No	499
				Italy	INGI-FVG	No	1432
				Italy	INGI-CILENTO	No	1147

				Germany	Cooperative Health Research in the Region of Augsburg (KORA) F3	Yes (controls)	1640
				Germany	KORA F4	Yes (controls)	1809
				Germany	LURIC Study	No	2927
				Sweden	the Prospective Investigation of the Vasculature in Uppsala Seniors (PIVUS)	No	945
				Germany	SHIP-Trend	Yes	986
				UK	TwinsUK		3965
	Magnesium	Serum Magnesium	Meyer[18]	USA	ARIC	No	8,122
				USA	FHS	No	2,866
				Netherlands	RS	Yes	4,378
	Phosphate	Serum Phosphorus	Kestenbaum[19]	USA	CHS	No	2,337
				USA	ARIC	No	8,122
				USA	FHS	No	2,865
				Netherlands	RS	Yes	3,516
Microminerals	Iron	Serum Iron & Ferritin	Benyamin[20]	Australia	Queensland Institute of medical research (QIMR)	Yes	11,692
				Estonia	Estonian Genome Project	No	893
				Italy	Val Borbera Study	No	1,659
				Netherlands	Nijmegen Biomedical Study	No	1,791
				UK	UK Blood Services Common Controls Panel	Yes (controls)	2,419
				Italy	Micros/ Eurac	No	1,218
				Netherlands	ERF/ Rotterdam	Yes	871
				Germany	Kora F3/ F4	Yes (controls)	3,443
				Australia	Busselton Health Study	No	877

			Estonia	Estonian Genome Project	No	1,017
			Italy	InCHIANTI	No	1,206
			Sardinia	SardiNIA study of Aging	No	4,694
			Switzerland	CoLaus	Yes	5,419
			Netherlands	Prevention of Renal and Vascular Endstage Disease (PREVEND)	No	3,644
			UK	Fenland	No	1,402
			France, Italy, Spain, UK, Netherlands, Germany, Sweden, Denmark	INTERACT	No	9,294
Copper	Erythrocyte Copper	Evans[21]	Australia	QIMR	Yes	2,603
Manganese	Serum Manganese	Ng[22]	Sweden	PIVUS	No	949
Selenium	Serum Selenium	Evans[21]	Australia	QIMR	Yes	2,603
			UK	Avon Longitudinal Study of Parents and Children (ALSPAC)	No	2,874
Zinc	Erythrocyte Zinc	Evans[21]	Australia	QIMR	Yes	2,603

Supplementary Table S4: Micronutrient instruments used in analyses

SNP lists used in primary analyses are listed for each micronutrient; N = sample size; Chr = Chromosome P = p value for association; SNPs were mapped to genes using the GWAS catalog metadata or derived from original publications. Details of gene function were obtained from the National Library of Medicine (NLM) Gene database (https://www.ncbi.nlm.nih.gov/gene/). Genes encoding proteins directly related to the metabolism of each micronutrient (defined as processes related to micronutrient uptake, synthesis, transport, or degradation) were included in 'functional' sensitivity analyses and postfixed with an asterix* in the gene column. Where the description given on the NLM Gene database did not clearly specify a physiological function with direct relevance to the metabolism of the specified micronutrient, biological pathways for the gene were cross-checked with the Kyoto Encyclopedia of Genes and Genomes (KEGG) pathway resource (https://www.kegg.jp/kegg/).[23]
Additional SNPs included in cIVW methods available on request from authors, or through the IEU Open GWAS https://gwas.mrcieu.ac.uk;
GWAS id from the IEU Open GWAS is given in brackets: Vitamin D (*ebi-a-GCST005367*), copper (*ieu-a-1073*), selenium (*ieu-a-1077*) and zinc (*ieu-a-1079*).

Micronutrient	*GWAS*	*N*	*SNP*	*P*	*Chr*	gene		*Function of gene with respect to micronutrient*
Vitamin A (serum retinol)	Mondul 2011	9302	rs10882272	6.51E-15	10	RBP4*	Retinol binding protein 4	Encodes retinol binding protein, which delivers retinol from the liver stores to the peripheral tissues
		9302	rs1667255	6.35E-14	18	TTR*	Transthyretin	Encodes transthyretin, which transports thyroid hormones in the plasma and cerebrospinal fluid. It is also involved in the transport of retinol (vitamin A) in the plasma by associating with retinol-binding protein.
Vitamin A (beta carotene)	Ferrucci 2009	3881	rs6564851	1.6E-24	16	BCO1*	Beta-carotene oxygenase 1	The protein encoded by this gene is a key enzyme in beta-carotene metabolism to vitamin A. It catalyzes the oxidative cleavage of beta,beta-carotene into two retinal molecules
Vitamin B6	Tanaka 2009	1,864	rs4654748	8.30E-18	1	ALPL	Alkaline Phophatase	This gene encodes a member of the alkaline phosphatase family of proteins. The product of this gene is a membrane bound glycosylated enzyme that is not expressed in any particular tissue and is, therefore, referred to as the tissue-nonspecific form of the enzyme
Folate	Grarup 2013	37,465	rs652197	1.40E-12	11	FOLR3*	Folate receptor gamma	Encodes a member of the folate receptor family of proteins, which mediate the delivery of 5-methylhydroolate to interior of cells
		37,337	rs1801133	9.50E-53	1	MTHFR*	Methylenetetrahydrofolate reductase	Encodes a protein that catalyses the conversion of 5,10,methylenetetrahydrofolate to 5-methyltetrahydrofolate
Homocysteine	Van Meurs 2013	44,147	rs1801133	4.34E-104	1	MTHFR	Methylenetetrahydrofolate reductase	Catalyzes the conversion of 5,10-methylenetetrahydrofolate to 5-methyltetrahydrofolate, a co-substrate for homocysteine remethylation to methionine
			rs2275565	1.96E-10	1	MTR	5-methyltetrahydrofolate-homocysteine methyltransferase	Catalyzes the remethylation of homocysteine to methionine
			rs9369898	2.17E-10	6	MMUT	Methylmalonyl-CoA mutase	A vitamin B12-dependent enzyme which catalyzes the isomerization of methylmalonyl-CoA to succinyl-CoA
			rs7130284	1.88E-20	11	NOX4	NADPH oxidase 4	An oxygen sensor that catalyzes the reduction of molecular oxygen to various reactive oxygen species.
			rs154657	1.74E-43	16	DPEP1	Dipeptidase 1	Kidney membrane enzyme that hydrolyzes a variety of dipeptides and is implicated in renal metabolism of glutathione and its conjugates
			rs234709	3.90E-24	21	CBS	Cystathione beta-synthase	Catalyzes the conversion of homocysteine to cystathionine, the first step in the trans-sulfuration pathway

			rs548987	1.12E-08	6	SLC17A3	Solute carrier family 17, member 3	The protein encoded by this gene is a voltage-driven transporter that excretes intracellular urate and organic anions from the blood into renal tubule cells.
			rs42648	1.97E-08	7	GTBP10	GTP binding protein 1	Small G proteins, such as GTPBP10, act as molecular switches that play crucial roles in the regulation of fundamental cellular processes such as protein synthesis, nuclear transport, membrane trafficking, and signal transduction
			rs1801222	8.43E-10	10	CUBN	Cubilin	A receptor for intrinsic factor-vitamin B12 complexes
			rs2251468	1.28E-12	12	HNF1A	HNF homeobox A	A transcription factor required for the expression of several liver-specific genes
			rs838133	7.48E-09	19	FUT2	Fucosyltransferase 2	This gene is one of two encoding the galactoside 2-L-fucosyltransferase enzyme. The encoded protein is important for the final step in the soluble ABO blood group antigen synthesis pathway. It is also involved in cell-cell interaction, cell surface expression, and cell proliferation.
			rs12780845	7.80E-10	10	CUBN	Cubilin	A receptor for intrinsic factor-vitamin B12 complexes
Vitamin B12	Grarup 2013	45,575	rs2336573	8.40E-59	19	CD320*	CD320 molecule	This gene encodes the transcobalamin receptor that is expressed at the cell surface. It mediates the cellular uptake of transcobalamin bound cobalamin (vitamin B12).
		45,575	rs1131603	4.90E-49	22	TCN2*	Transcobalamin 2	This gene encodes a member of the vitamin B12-binding protein family. This plasma protein binds cobalamin and mediates the transport of cobalamin into cells.
		45,571	rs3742801	1.70E-13	14	ABCD4*	ATP binding cassette subfamily D member 4	The protein encoded by this gene is a member of the superfamily of ATP-binding cassette (ABC) transporters. Lysosomal membrane protein that transports cobalamin (Vitamin B12) from the lysosomal lumen to the cytosol in an ATP-dependent manner
		45,576	rs2270655	2.20E-13	4	MMAA*	Metabolism of cobalamin associated A	Involved in translocation of vitamin B-12 into the mitochondria
		45,576	rs34324219	1.10E-111	11	TCN1*	Transcobalamin 1	This gene encodes a member of the vitamin B12-binding protein family, which facilitates the transport of cobalamin into cells.
		45,575	rs7788053	1.70E-10	19	FUT6	Fucosyltransferase 6	The protein encoded by this gene is a Golgi stack membrane protein
		45,568	rs602662	2.40E-139	19	FUT2	Fucosyltransferase 2	As above
		45,576	rs1801222	3.30E-75	10	CUBN*	Cubilin	Cubilin (CUBN) acts as a receptor for intrinsic factor-vitamin B12 complexes. Cubulin is located within the epithelium of intestine and kidney.

		45,576	rs41281112	8.90E-35	13	CLYBL*	Citramalyl-CoA lyase	Enables (S)-citramalyl-CoA lyase activity; magnesium ion binding activity; and malate synthase activity. Involved in protein homotrimerization and regulation of cobalamin metabolic process.
		45,574	rs1141321	3.60E-26	6	MMUT	Methylmalonyl-CoA mutase	Vitamin B-12 dependent enzyme
Vitamin C	Zheng 2021	52,018	rs6693447	6.25E-10	1	RER1	Rhodopsin enhancer region	A conserved regulatory region upstream of the rhodopsin (RHO) gene, thought to bind retina-specific transcription factors
			rs13028225	2.38E-30	2	SLC23A3	Solute carrier family 23 member 3	Predicted to enable transmembrane transporter activity.
			rs33972313	4.61E-90	5	SLC23A1*	Solute carrier family 23 member 1	Encodes one of the two sodium-dependent vitamin C transporters used to transport vitamin C into the body and its distribution to organs
			rs10051765	3.64E-09	5	RGS14	Regulator of G protein signalling	Encodes a member of the regulator of G-protein signaling family
			rs7740812	1.88E-09	6	GSTA5	Glutathione S-transferase alpha 5	Encodes glutathione S-transferase (GST)-α5, which catalyzes the conjugation of reduced glutathiones and a variety of electrophiles
			rs174547	3.84E-08	11	FADS1	Fatty acid Desaturase 1	Encodes fatty acid desaturase enzyme.
			rs117885456	1.70E-11	12	SNRPF	small nuclear ribonucleoprotein polypeptide F	Unclear relevance to vitamin C metabolism
			rs2559850	6.30E-20	12	CHPT1	Choline phosphotransferase 1	Enables diacylglycerol cholinephosphotransferase activity.
			rs10136000	1.33E-08	14	AKT1	AKT serine/threonine kinase 1	Encodes AKT serine-thronine protein kinase family.
			rs56738967	7.62E-10	16	MAF	MAF bZIP transcription factor	The protein encoded by this gene is a DNA-binding, leucine zipper-containing transcription factor
Vitamin D	Jiang 2018	42,274	rs3755967	4.74e-343	4	GC*	GC vitamin binding protein	The protein encoded by this gene binds to vitamin D and its plasma metabolites and transports them to target tissues.
			rs10741657	2.05E-46	11	CYP2R1*	Cytochrome P450 family 2 subfamily R member 1	Encodes and enzyme that catalyzes vitamin D into the active form
			rs12785878	3.8E-62	11	NADSYN1/DHCR7*	Cytochrome P450 family 2 subfamily R member 1	This enzyme is a microsomal vitamin D hydroxylase that converts vitamin D into the active ligand for the vitamin D receptor.
			rs10745742	1.88E-14	12	AMDHD1	amidohydrolase domain containing 1	Predicted to enable imidazolonepropionase activity. Predicted to be involved in histidine catabolic process.
			rs8018720	4.72E-09	14	SEC23A	SEC23 homolog A	Encodes a protein found in the ribosome-free transitional face of the endoplasmic reticulum (ER) and associated vesicles.

			rs17216707	8.14E-23	20	CYP24A1*	Cytochrome p450 family 24 subfamily A member 1	Mitochondrial protein that initiates the degradation of the physiologically active form (hormonal form) of vitamin D, playing a role in calcium and vitamin D homeostasis
Calcium	O'Seagh ada 2013	61,054	rs1801725	8.90E-86	3	CASR*	Calcium sensing receptor	Encodes a membrane G coupled calcium sensing receptor that senses small changes in calcium and maintains homeostasis through intracellular signalling pathways and parathyroid hormone secretion
		60,958	rs780094	1.30E-10	2	GCKR	Glucokinase regulator	Encodes glucokinase regulator, which plays a role in glucose homeostasis, but has multiple metabolic roles including of liver enzymes, amino acids and serum albumin
		60,040	rs10491003	4.80E-09	10	GATA3	GATA binding protein 3	GATA3 encodes a transcription factor involved in parathyroid gland development, therefore calcium homeostasis
		61,011	rs7481584	1.20E-10	11	CARS1	Cysteinyl-tRNA synthetase	Encodes a cysteinyl-tRNA synthetase. Variants in this region lead to Beckwith-Wiedmann syndrome, associated with hypocalcemia and hypercalciuria
		60,928	rs7336933	9.10E-10	13	DGKH/KIAA0 564	Diacylglycerol kinase eta	Encodes diacylglycerol kinase eta, which is involved in regulating intracellular diacylglycerol and phophatidic acid. Variants in this gene have been associated with bipolar disorder
		60,966	rs1570669	9.10E-12	20	CYP24A1	Cytochrome p450 family 24 subfamily A member 1	Mitochondrial protein that initiates the degradation of the physiologically active form (hormonal form) of vitamin D, playing a role in calcium and vitamin D homeostasis
Copper	Evans 2013	2,603	rs1175550	5.03E-10	1	SMIM1	small integral membrane protein 1	Encodes a protein involved in red blood cell formation
			rs2769264	2.63E-20	1	SELENBP1	selenium binding protein 1	Encodes selenium binding protein. Uncertain role in copper metabolism
Ferritin	Benyam in 2014	48,972	rs1800562	1.54E-38	6	HFE	Homeostatic iron regulator	Encodes a protein that regulates iron absorption. Hereditary haemochromatosis is a recessive disorder resulting from defects in this gene
			rs855791	1.38E-14	22	TMPRSS6	Transmembrane serine protease 6	The protein encoded by this gene is a type II transmembrane serine proteinase that is found attached to the cell surface.
			rs744653	8.37E-19	2	SLC40A1	Solute carrier family 40 member 1	SLC40A1 is a cell membrane protein involved in iron export from duodenal epithelial cells. Defects in SLC40A1 are a cause of hemochromatosis type 4
			rs651007	1.31E-08	9	ABO	alpha 1-3-galactosyltransferase	Encodes proteins related to ABO blood system

			rs411988	1.59E-10	17	TEX14	Testis expressed 14, intracellular bridge forming factor	Encodes protein necessary for intercellular bridges in germ cells, which are required for spermatogenesis. Unclear relevance for iron homeostasis.
Iron	Benyam in 2014	48,972	rs1800562	2.72E-97	6	HFE*	Homeostatic iron regulator	As above
			rs1799945	1.10E-81	6	HFE*	Homeostatic iron regulator	As above
			rs855791	1.32E-139	22	TMPRSS6	Transmembrane serine protease 6	As above
			rs8177240	6.65E-20	3	TF*	Transferrin	As above
			rs7385804	1.36E-18	7	TFR2*	Transferrin receptor 2	As above
Magnesium	Meyer 2010	15,366	rs4072037	2.01E-36	1	MUC1	Mucin 1, cell surface associated	Unclear relevance for magnesium homeostasis. Involved in forming protective mucous barriers on epithelial surfaces, and have a role in intracellular signaling
			rs13146355	6.27E-13	4	SHROOM3	Shroom family member 3	Encodes protein that may regulate cell types in certain tissues. Involved in neural tube closure
			rs11144134	8.21E-15	9	TRPM6*	Transient receptor potential cation channel subfamily M member 6	Encodes a TRP ion channel subunit which mediates magnesium reuptake in kidneys
			rs3925584	5.20E-16	11	DCDC5	doublecortin domain containing 5	Unclear relevance for magnesium physiology
			rs448378	1.25E-08	3	MDS1	Myelodysplasia syndrome 1	Unclear relevance for magnesium physiology
Manganese	Ng 2015	949	rs1776029	2.17E-14	1	SLC30A10*	Solute carrier family 30 member 10	This gene plays a critical role in manganese transport and is induced by manganese in the liver
			rs13107325	5.08E-11	4	SLC39A8*	Solute carrier family 39 member 8	Encodes a protein found in plasma membrane and mitochondria involved in the cellular transport of zinc and manganese
Phosphorus	Kestenbaum 2010	21,726	rs1697421	3.47E-16	1	ALPL*	Alkaline Phosphatase	Encodes a membrane bound enzyme that hydrolyses pyrophosphate into phosphate in cells. Rare mutations in this gene linked to hypophophatasia with failure to mineralize teeth and bone
			rs17265703	6.26E-08	3	CSTA	Cystatin A	Unclear relevance
			rs9469578	5.15E-10	6	IHPK3	Inositol hexakisphosphate kinase 3	Encodes a protein that belongs to the inositol phosphokinase (IPK) family. This protein is likely responsible for the conversion of inositol hexakisphosphate (InsP6) to diphosphoinositol pentakisphosphate (InsP7/PP-InsP5).
			rs947583	2.19E-09	6	PDE7B	Phosphodiesterase 7B	Hydrolyzes cAMP, which regulates many important physiological processes. May be involved in the control of cAMP-mediated neural activity and cAMP

								metabolism in the brain. Gene variants appear associated with Dyslexia
			rs2970818	4.04E-08	12	FGF6	Fibroblast growth factor 6	Plays an important role in the regulation of cell proliferation, cell differentiation, angiogenesis and myogenesis, and is required for normal muscle regeneration
Selenium	Evans 2013	2,603	rs921943	9.40E-28	5	DMGDH/ BHMT2	Dimethylglycine dehydrogenase/ betaine-homocysteine S-methyltransferase 2	Exact relevance to selenium status unclear. DMGDH encodes enzymes involved in choline catabolism. BHMT2 is one of two methyl transferases that can catalyze the transfer of the methyl group from betaine to homocysteine.
Zinc	Evans 2013	2603	rs1532423	6.40E-12	8	CA1	Carbonic anhydrase 1	Carbonic Anhydrases are a large family of zinc containing enzymes that catalyse the reversible hydration of carbon dioxide
			rs2120019	1.55E-18	15	PPCDC	Phosphopantothenoylcysteine decarboxylase	Protein coding gene involved in biosynthesis of coenzyme A (CoA) from vitamin B5, involved in multiple metabolic processes.

Supplementary Table S5: Heterogeneity Statistics for MR analyses

Heterogeneity statistics for each MR analysis is given below: number of SNPs in analysis (nSNPs); percentage of variance explained in exposure for primary analyses (R^2), mean F statistic (mFc); I2 statistic (I2), Cochran's Q, Rucker's Q and Egger intercept, with p values in brackets. clumping threshold (r^2, either 0.001 for traditional analyses, and 0.2 for correlated analyses). As none of the analyses had SNPs removed after Steiger filtering, the results are equivalent.

							MAJOR DEPRESSIVE DISORDER			RECURRENT DEPRESSION		
		**nSNPs**	**R^2**	**r^2**	**mFc**	**I^2**	**Cochrans Q (p)**	**Ruckers Q (p)**	**Egger Int (p)**	**Cochrans.Q.p.**	**Ruckers.Q.p.**	**Egger.Intercept..p.**
Water Soluble Vitamins	**Vitamin B6**	1	1.5%	NA	27	NaN	NA (NA)	NA (NA)	NA (NA)	NA (NA)	NA (NA)	NA (NA)
	**Vitamin B9**											
	Folate	2	0.6%	0.001	620	0.94	NA (NA)	NA (NA)	NA (NA)	NA (NA)	NA (NA)	NA (NA)
	Homocysteine	12	2.8%	0.001	101	0.99	29 (0.003)	28 (0.002)	-0.002 (0.70)	13 (0.32)	12 (0.26)	-0.004 (0.81)
	**Vitamin B12**	9	5.4%	0.001	192	0.98	17 (0.03)	17 (0.02)	0.0028 (0.73)	10 (0.25)	7 (0.47)	0.03 (0.1)
	Functional	*7*	3.7%	0.001	159	0.98	16 (0.01)	16 (0.01)	0.005 (0.69)	7 (0.28)	3 (0.76)	0.04 (0.08)
	**VitaminC**	11	1.8%	0.001	85	0.97	21 (0.02)	14 (0.12)	0.008 (0.06)	20 (0.026)	20 (0.016)	1.3e-05 (1)
	Functional	*1*	0.8%	NA	400	NaN	NA (NA)	NA (NA)	NA (NA)	NA (NA)	NA (NA)	NA (NA)
Fat soluble vitamins	***Vitamin A***											
	Retinol	2	0.1%	0.001	56	0.99	NA (NA)	NA (NA)	NA (NA)	NA (NA)	NA (NA)	NA (NA)
	Beta carotene	1	1.1%	0.001	99	NaN	NA (NA)	NA (NA)	NA (NA)	NA (NA)	NA (NA)	NA (NA)
	**Vitamin D**	6	3.6%	0.001	169	0.98	2 (0.8)	2 (0.68)	-0.0003 (0.95)	2 (0.79)	2 (0.68)	0.004 (0.79)
	Functional	*4*	3.4%	0.001	234	0.99	2 (0.54)	2 (0.38)	-0.003 (0.69)	1 (0.69)	1 (0.5)	-0.006 (0.8)
	Correlated	*29*		0.2	296		44 (0.03)	31 (0.27)	1.01 (<0.001)	35 (0.16)	35 (0.14)	1 (0.47)

	Serum Calcium	*7*	*0.2%*	*0.001*	*74*	*0.96*	*23 (0.001)*	*18 (0.003)*	*-0.01 (0.27)*	*9 (0.17)*	*8 (0.14)*	*0.01 (0.52)*
	Functional	*1*	0.1%	0.001	315	NaN	NA (NA)	NA (NA)	NA (NA)	NA (NA)	NA (NA)	NA (NA)
	**Magnesium**	5	2.2%	0.001	60	0.9	9 (0.07)	5 (0.14)	-0.01 (0.27)	7 (0.15)	4 (0.24)	-0.04 (0.27)
	Functional	*1*	0.4%	NA	121	NaN	NA (NA)	NA (NA)	NA (NA)	NA (NA)	NA (NA)	NA (NA)
	Correlated	*7*		0.2	48		10 (0.15)	9 (0.11)	0.99 (0.56)	7 (0.31)	5 (0.42)	0.96 (0.14)
Macrominerals												
	**Phosphate**	5	1.2%	0.001	40	0.98	4 (0.44)	4 (0.29)	-0.003 (0.87)	4 (0.45)	4 (0.32)	-0.02 (0.75)
	Functional	*1*	0.4%	0.2	67	NaN	NA (NA)	NA (NA)	NA (NA)	NA (NA)	NA (NA)	NA (NA)
	**Copper**	2	5.3%	0.001	62	0.99	NA (NA)	NA (NA)	NA (NA)	NA (NA)	NA (NA)	NA (NA)
				0.2	378		4 (0.66)	4 (0.54)	1 (0.98)	4 (0.64)	4 (0.59)	0.98 (0.46)
	**Ferritin**	6	1.2%	0.001	69	0.98	5 (0.41)	2 (0.67)	0.01 (0.18)	4 (0.57)	4 (0.42)	0.001 (0.94)
	Correlated	*6*		0.2	41		5 (0.37)	4 (0.47)	1.01 (0.18)	4 (0.51)	4 (0.37)	1 (0.81)
	**Iron**	4	3.3%	0.001	315	1	4 (0.22)	4 (0.13)	-0.003 (0.70)	1 (0.72)	0 (0.79)	-0.02 (0.45)
	Functional	*4*	2.6%	0.001	237	1	4 (0.28)	3 (0.18)	-0.003 (0.67)	1 (0.82)	1 (0.77)	-0.01 (0.60)
	Correlated	*31*		0.2	140		101 (<0.001)	101 (7.5e-10)	1 (0.79)	34 (0.29)	34 (0.25)	1 (0.72)
	**Manganese**	2	11.0%	0.001	50	0.81	NA (NA)	NA (NA)	NA (NA)	NA (NA)	NA (NA)	NA (NA)
	**Selenium**	1	2.5%	NA	119	NaN	NA (NA)	NA (NA)	NA (NA)	NA (NA)	NA (NA)	NA (NA)
	Correlated	*7*		0.2	109		4 (0.55)	1 (0.85)	1.02 (0.10)	3 (0.63)	3 (0.55)	1.03 (0.54)
Microminerals												
	**Zinc**	2	4.2%	0.001	61	0.99	NA (NA)	NA (NA)	NA (NA)	NA (NA)	NA (NA)	NA (NA)
	Correlated	*7*		0.2	225		16 (0.01)	15 (0.01)	1 (0.59)	7 (0.32)	5 (0.43)	0.98 (0.14)

Supplementary Table S6: Table containing SE_{IVW}/SE_{cIVW} ratios for relevant micronutrients.

The table below provides ratios of standard errors for each micronutrient/ outcome set. MR estimates derived using correlated MR methods should not be 'strikingly more precise' than the estimates derived using uncorrelated variants.[24] Correlated MR estimates with standard errors 2-3 times smaller than the estimate using non-correlated instruments should be checked carefully. In particular, as estimates are highly dependent on the LD matrix, this must reflect the population ancestry of the exposure/ outcome GWAS samples.[24] The harmonized exposure/ outcome alleles also need to be aligned with the alleles in the LD matrix as this can also bias results. Although the precision for several of the analyses was much higher than for the uncorrelated analyses, the consistency between effect estimates between traditional and correlated MR is reassuring.

	Major Depressive Disorder (n=430,775)	*Recurrent Depression (n= 80,933)*
	SE_{IVW} / SE_{cIVW}	*SE_{IVW} / SE_{cIVW}*
Vitamin D	1.59	2.36
Iron	1.58	2.03
Ferritin	1.31	0.96
Magnesium	1.13	1.44
Copper	2.63	3.06
Selenium	2.32	2.63
Zinc	1.16	3.38

Supplementary Table S7: Heterogeneity analyses for reverse MR analyses

Heterogeneity statistics for reverse MR analyses, in which MDD is treated as the exposure, and the micronutrient as the outcome. Results on the left are prior to Steiger filtering, with the results on the right after Steiger filtering. The clumping threshold for traditional analyses (r^2) is 0.001, but for cIVW this is relaxed to 0.2. The columns include number of SNPs in analysis (nSNPs); mean F statistic (mFc); percentage of variance explained in exposure (R^2), I2 statistic (I2), Cochran's Q, Rucker's Q and Egger intercept, with p values in brackets. For cEgger analyses, obly intercept p values are given in the R readout, and hence the intercept value is not included.

	Unfiltered								*Steiger Filtered*						
Outcome	**r^2**	**nSNPs**	**mFc**	**R^2**	**I2**	**Cochrans Q(p)**	**Ruckers Q(p)**	**Int (p)**	**nSNPs**	**mFc**	**R^2**	**I2**	**Cochrans Q(p)**	**Ruckers Q(p)**	**Int (p)**
Vitamin D	<0.001	66	45	*1.8%*	0.98	156 (0.002)	156 (0.002)	-6E-04 (0.77)	53	47	*1.5%*	0.98	37(0.94)	37(0.93)	0.001 (0.55)
	<0.2	110	43	*2.9%*	0.98	189 ($3E^{-6}$)	188 ($3E^{-6}$)	(0.41)	91	44	*2.4%*	0.98	103 (0.17)	102 (0.2)	(0.47)
Magnesium	<0.001	42	45	*1.20%*	0.98	41(0.47)	40(0.47)	-0.01(0.32)	26	44	0.70%	0.98	5(1)	5(1)	0.01 (0.65)
	<0.2	60	43	1.60%	0.98	73 (0.1)	72 (0.1)	(0.31)	36	43	0.9	0.98	8 (1)	8 (1)	(0.91)
Copper	<0.001	66	45	1.8%	0.98	50(0.92)	50(0.91)	-0.003 (0.90)	15	43	0.4%	0.98	0(1)	0(1)	-0.02 (0.78)
	<0.2	110	43	2.90%	0.98	143 (0.02)	141 (0.02)	(0.02)	27	41	0.70%	0.98	1(1)	1(1)	(0.79)
Iron	<0.001	64	46	1.8%	0.98	57(0.65)	57(0.67)	-0.01 (0.21)	46	45	1.3%	0.98	17(1)	17(1)	0.004 (0.74)
	<0.2	107	43	2.8%	0.98	165 ($2E^{-4}$)	163 ($3E^{-4}$)	(0.15)	71	43	1.9%	0.98	29 (1)	29 (1)	(0.75)
Ferritin	<0.001	64	46	1.8%	0.98	56(0.71)	52(0.81)	-0.02 (0.04)	48	45	1.3%	0.98	11(1)	11(1)	-0.004 (0.67)
	<0.2	104	43	2.70%	0.98	157 ($5E^{-4}$)	145 (0.004)	(0.001)	67	43	1.80%	0.98	19(1)	19(1)	(0.87)
Selenium	<0.001	66	45	*1.8%*	0.98	66 (0.45)	65 (0.44)	-0.023 (0.34)	18	45	0.5%	0.98	1 (1)	1 (1)	-0.01 (0.85)
	<0.2	110	43	2.9%	0.98	172 ($1E^{-4}$)	170 ($4E^{-4}$)	(0.18)	29	44	0.8%	0.98	1 (1)	1 (1)	(0.98)

Supplementary Material
Supplementary Material S1: Supplementary Methods

Calculating variance (r2)
Variance explained in the exposure was calculated for each SNP using the formula:

$$2 \times beta^2 \times EAF \times (1\text{-}EAF) \;/\; (2 \times beta^2 \times EAF \times (1\text{-}EAF) + SE^2 \times 2 \times N \times EAF \times (1\text{-}EAF))$$

Where beta is the effect size of the SNP on the exposure, and SE is the corresponding standard error, EAF is the effect allele frequency and N is the sample size of the exposure GWAS. The variance for each SNP in the instrument was summed to provide the total variance overall for that instrument set.

For analyses using depression as an exposure (ie reverse MR), we used the TwoSampleMR package to approximate the R-squared statistic using the "get_r_from_lor" function, with prevalence set at 10%. This result was squared to get the approximate r^2.

Calculating F Statistics
F-statistics for each exposure were calculated using the formula:

$$F= (\Sigma\, r^2/nSNP)/\; ((1\text{-}\Sigma\, r^2)/(N\text{-}nSNP\text{-}1))$$

Where $\Sigma\; r^2$ is the sum of the variance explained in the exposure for each SNP, nSNP is the number of SNPs and N is the sample size of the exposure GWAS

Supplementary Material S2: Individual Micronutrient MR plots

For each exposure, the following plots are shown:

i) Scatter plot showing how MR estimates compare between MR methods.

ii) Funnel plot depicting instrumental variable precision. The log(odds ratios) of each IV is plotted on the x-axis (β_{IV}) against instrument strength on the y axis ($1/SE_{IV}$). Asymmetry may suggest directional pleiotropy.

iii) Forest plot showing individual SNP ratio estimates (SNP-outcome estimate / SNP-exposure estimate), and

iv) Leave one out plot showing inverse variance weighted (IVW) estimates after omitting each SNP

Water Soluble Vitamins

Serum Folate and Major Depressive Disorder (N=430,775)

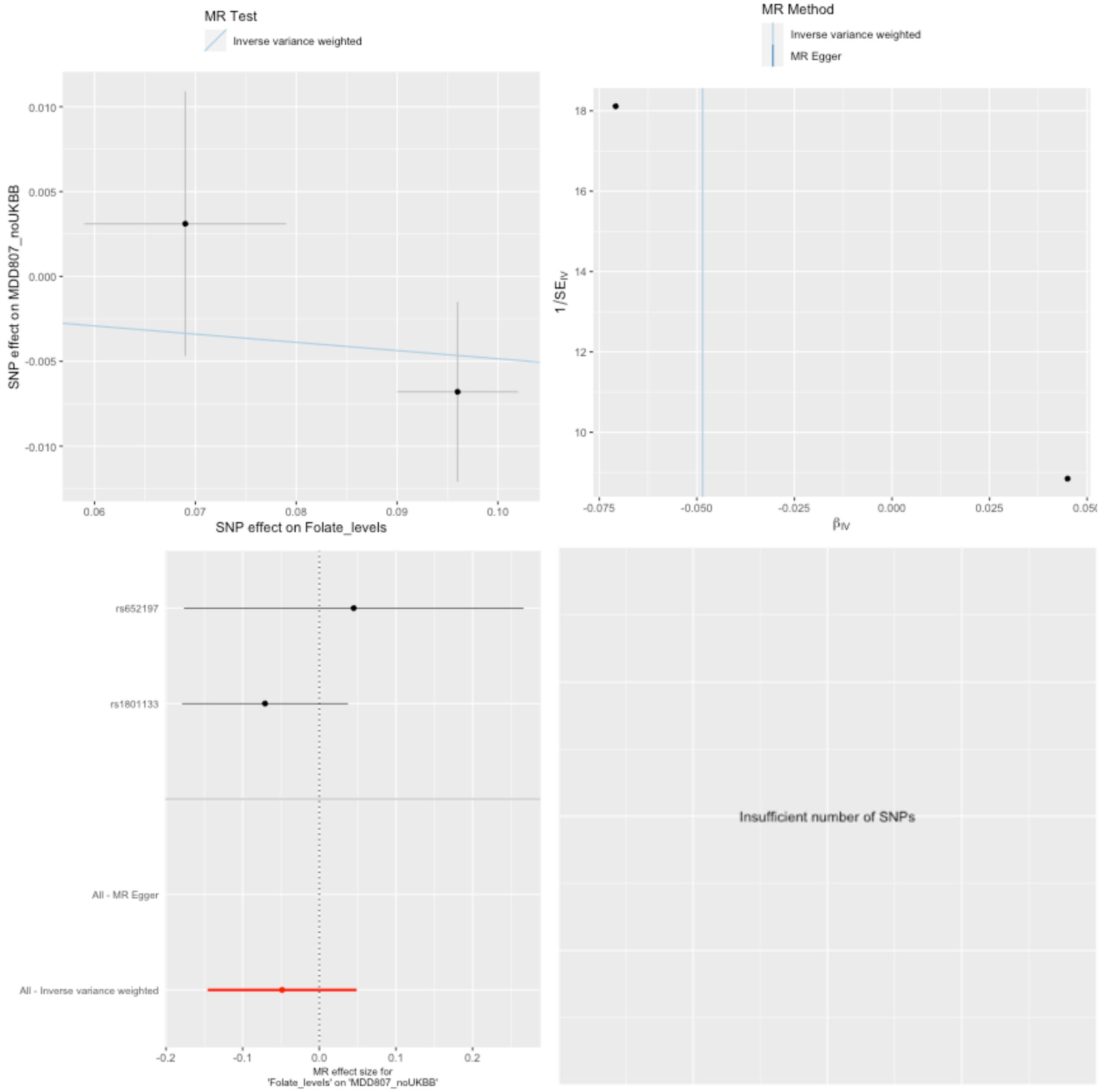

Serum Folate and Recurrent Depression (N= 80,933)

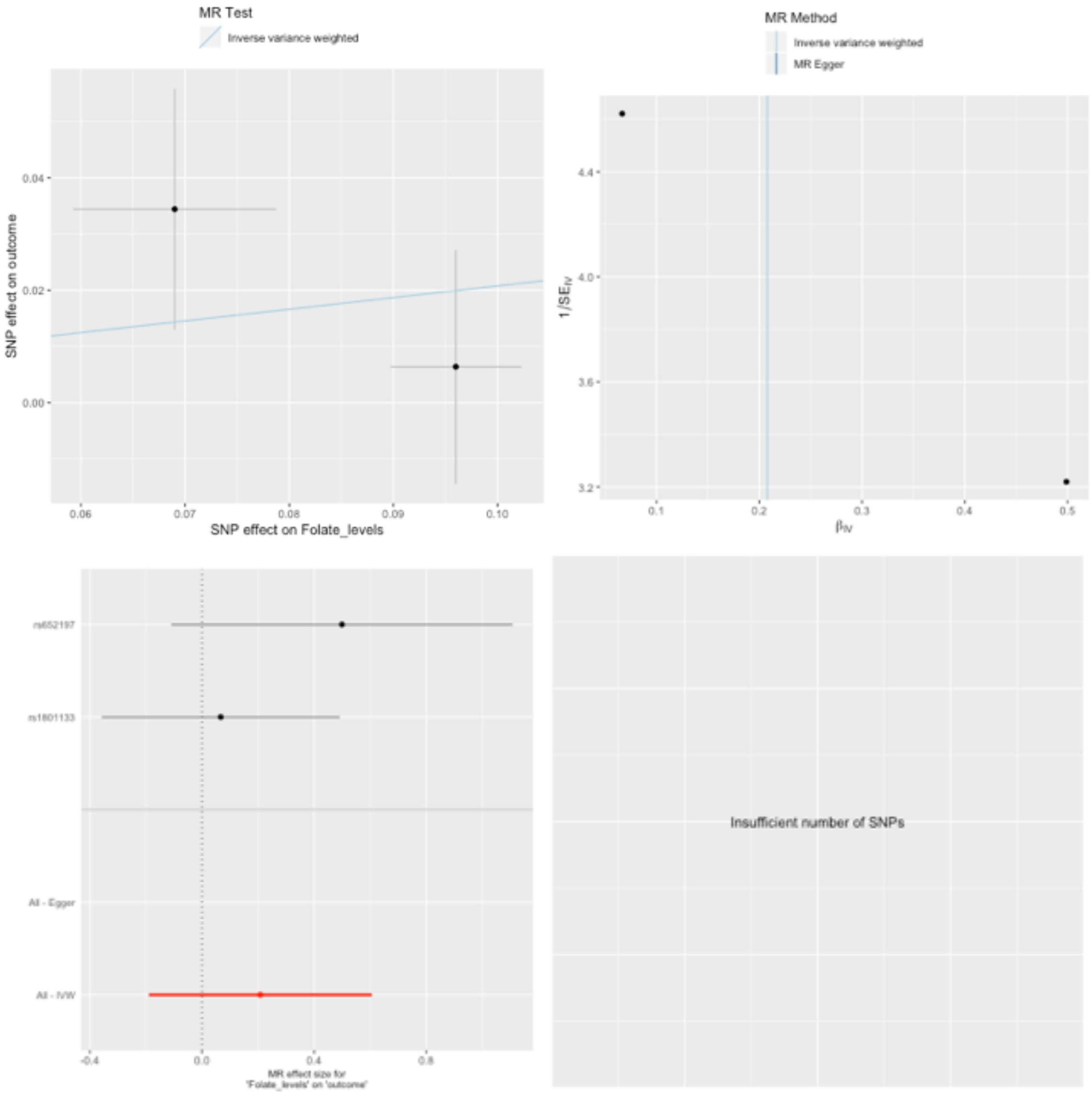

Serum homocysteine and Major Depressive Disorder (N=430,775)

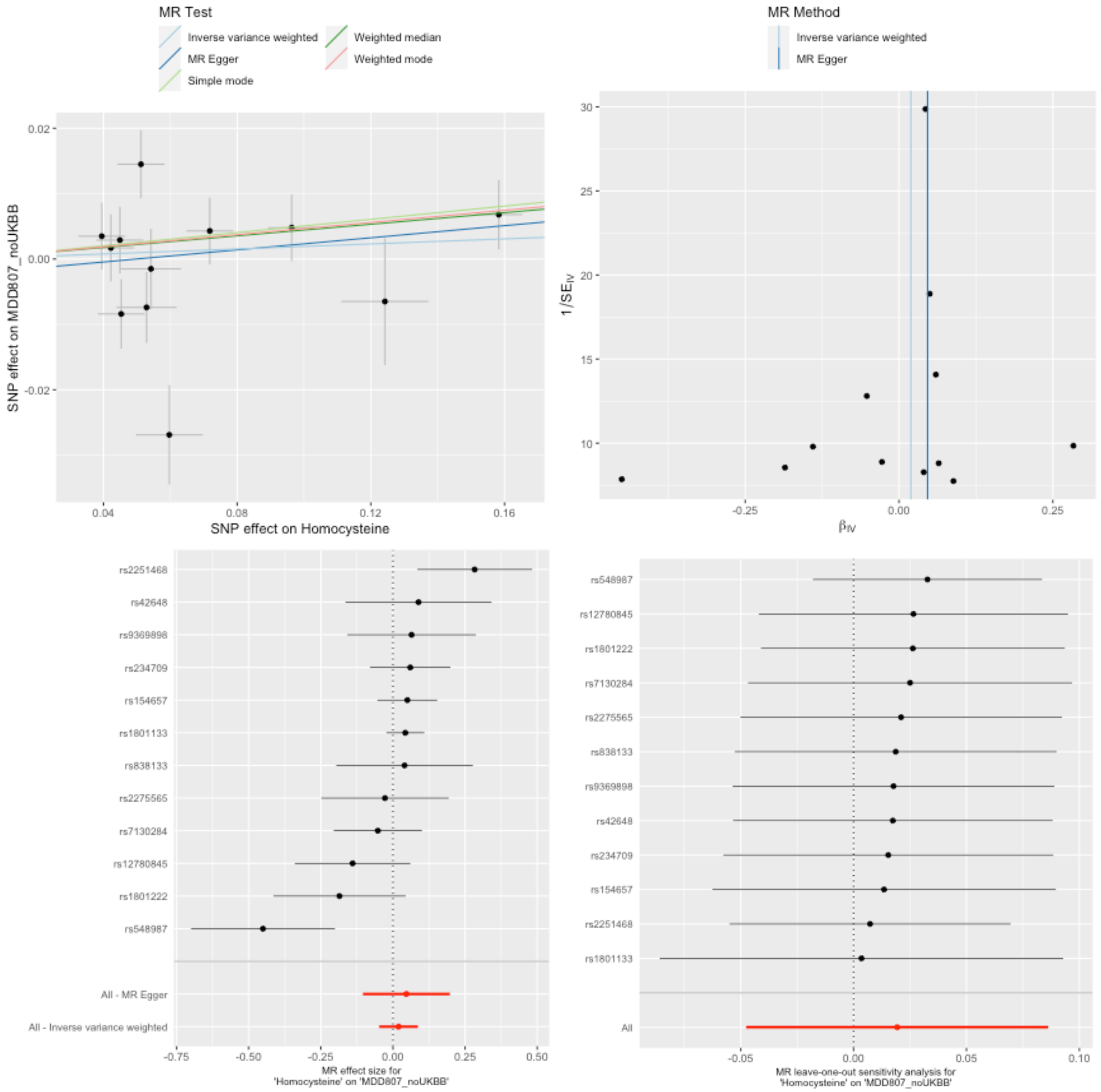

Serum Homocysteine and Recurrent Depression (N= 80,933)

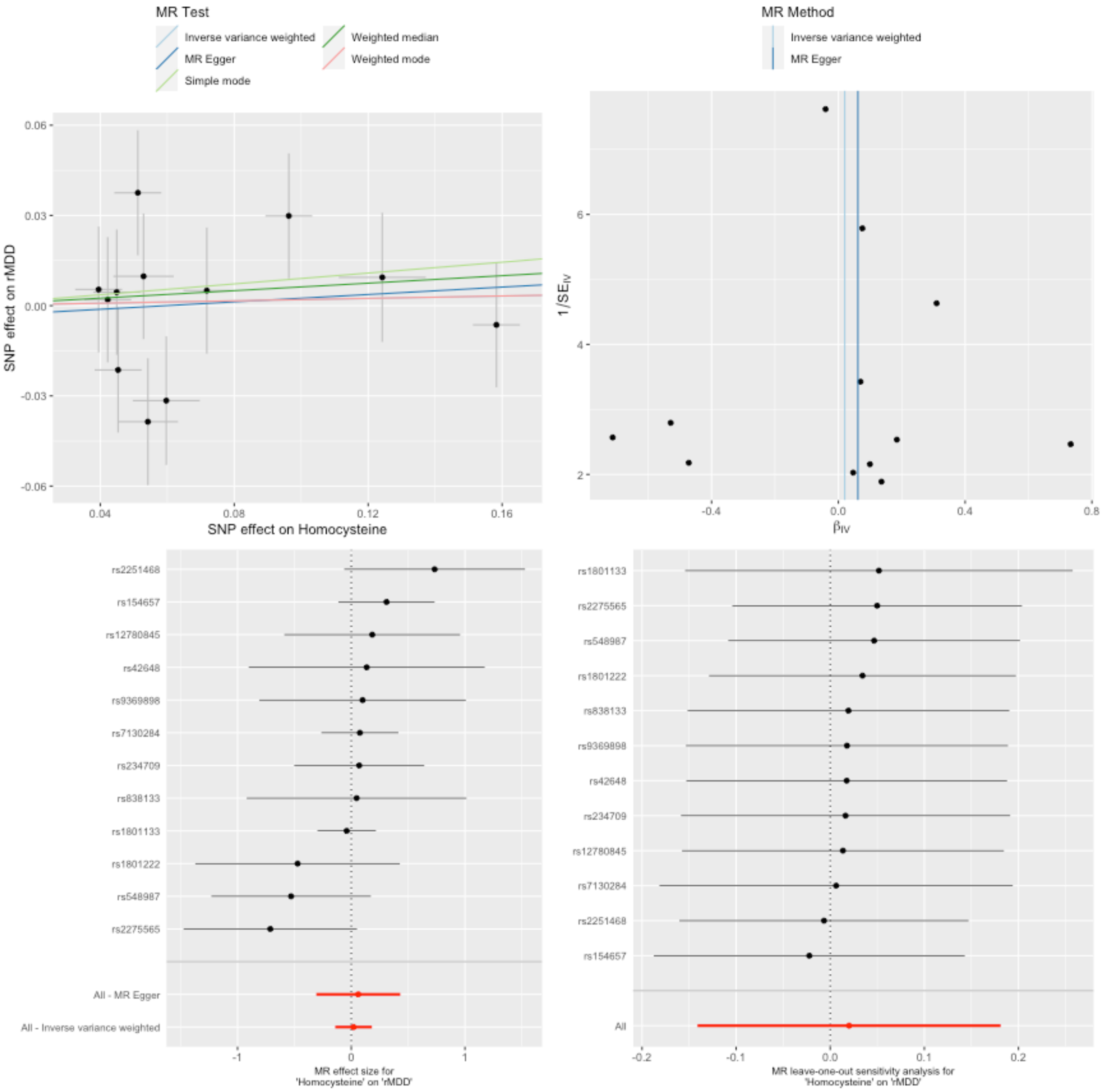

Serum B12 and Major Depressive Disorder (N=430,775)

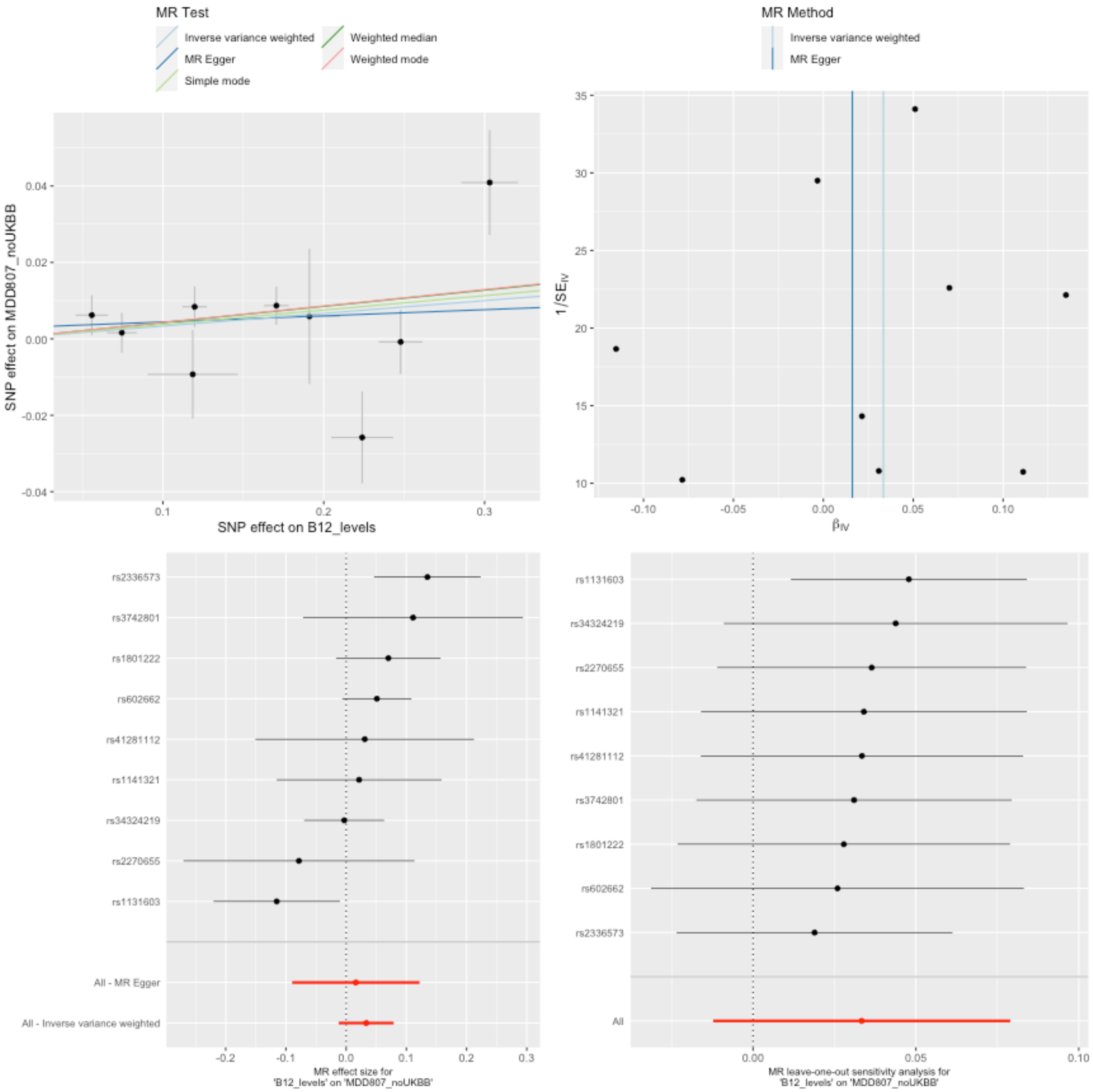

Serum B12 and Recurrent Depression (N= 80,933)

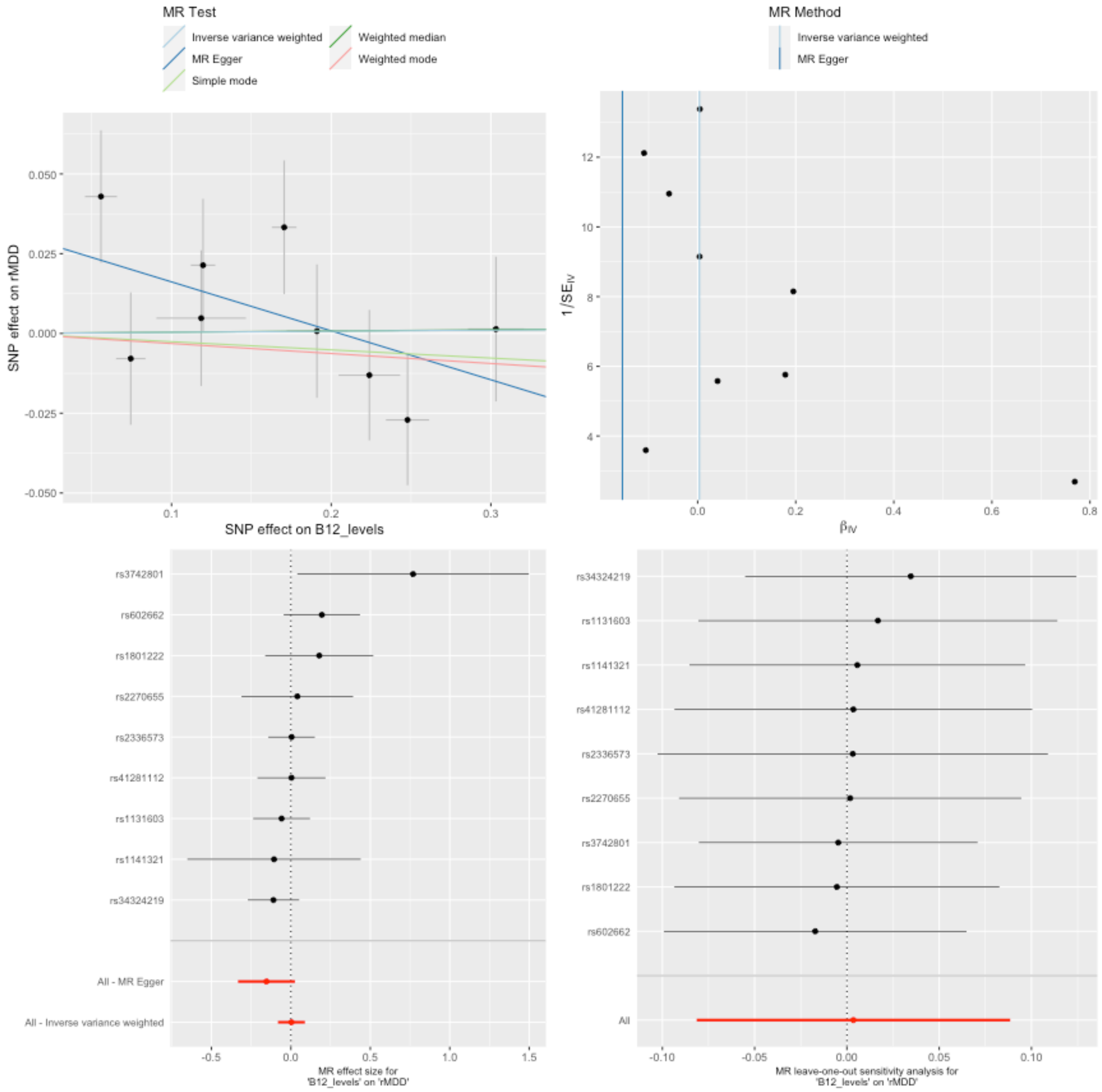

Vitamin C and Major Depressive Disorder (N=430,775)

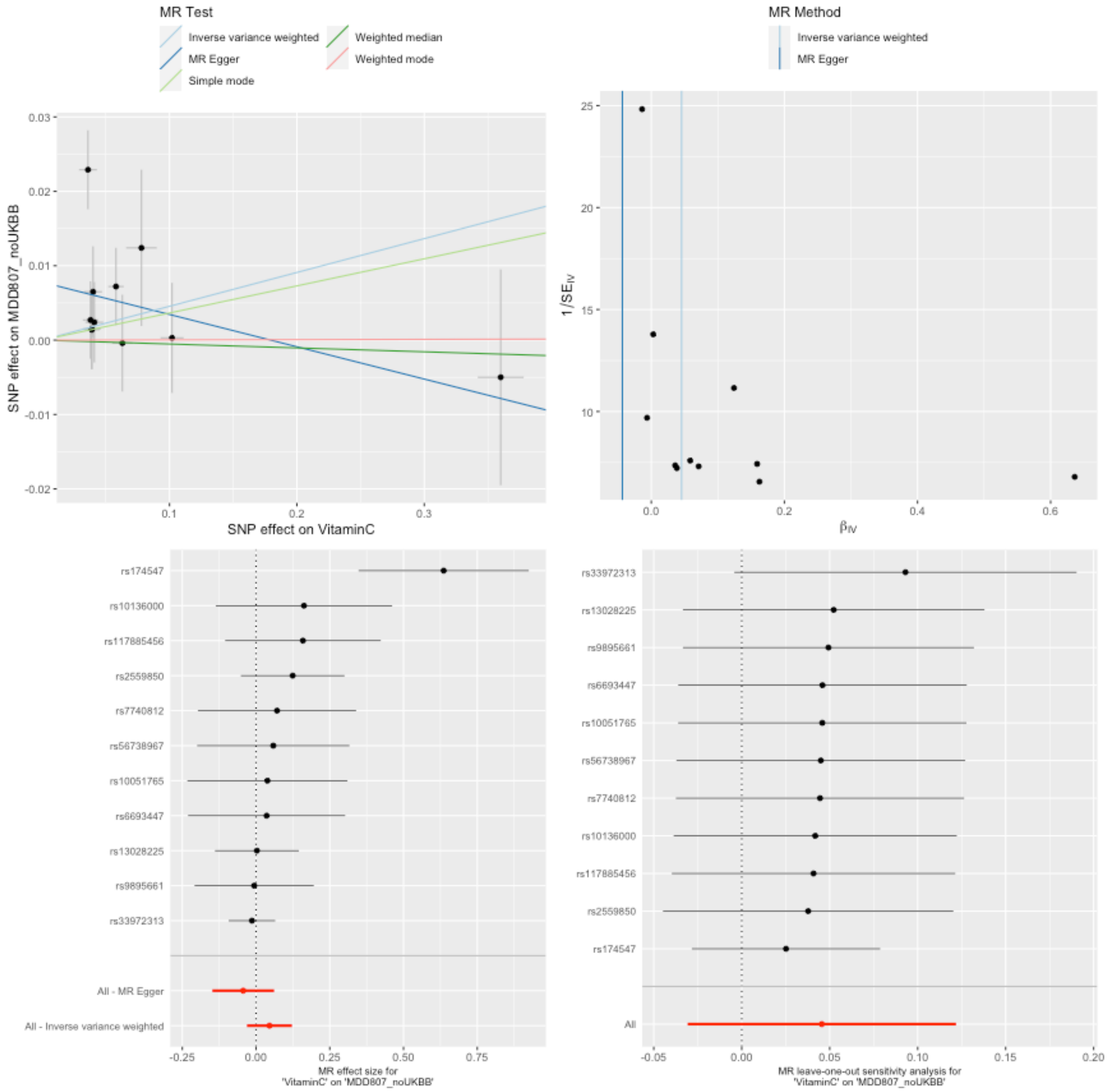

Vitamin C and Recurrent Depression (N= 80,933)

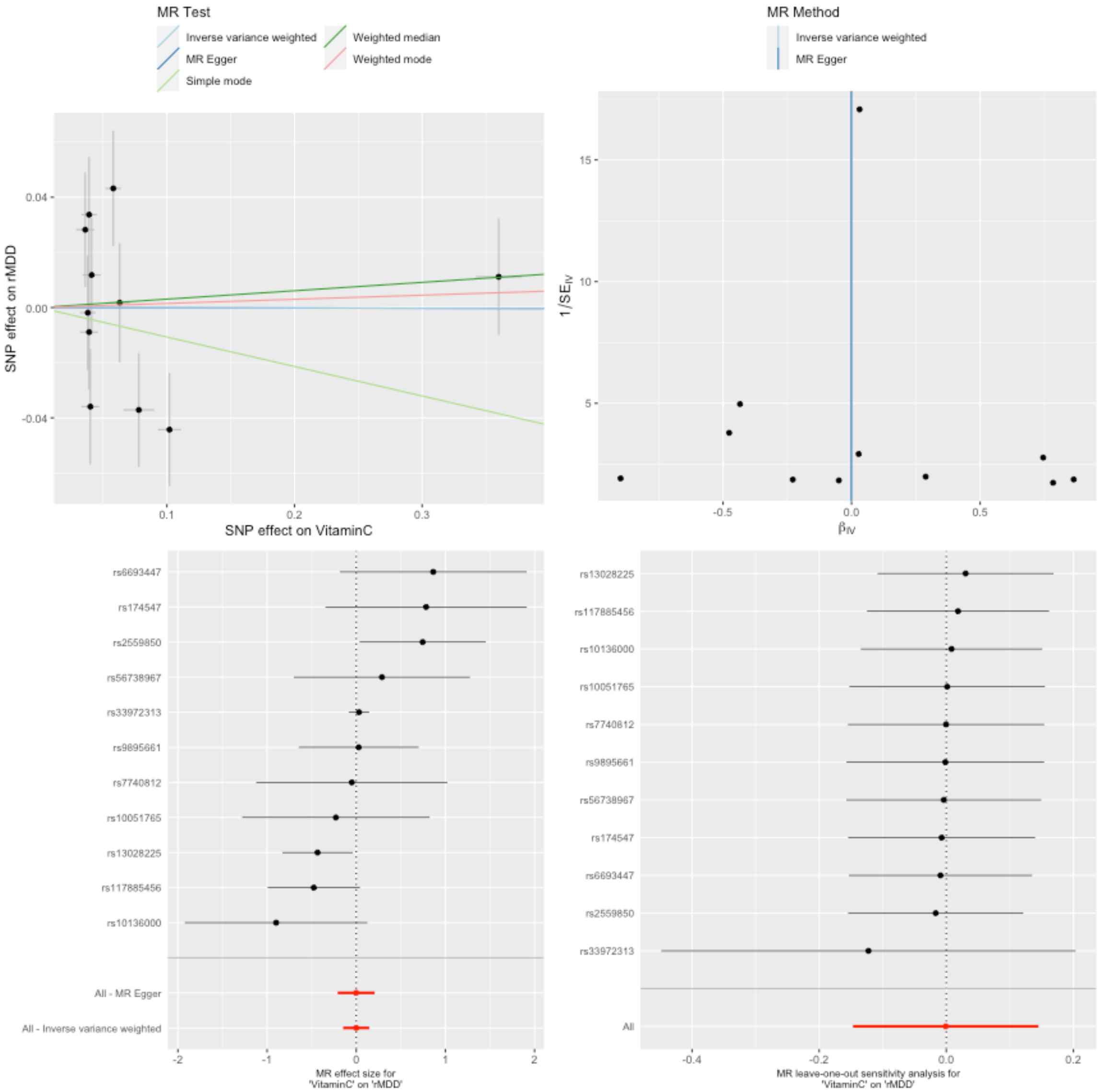

Fat Soluble Vitamins

Serum Retinol and Major Depressive Disorder (N=430,775)

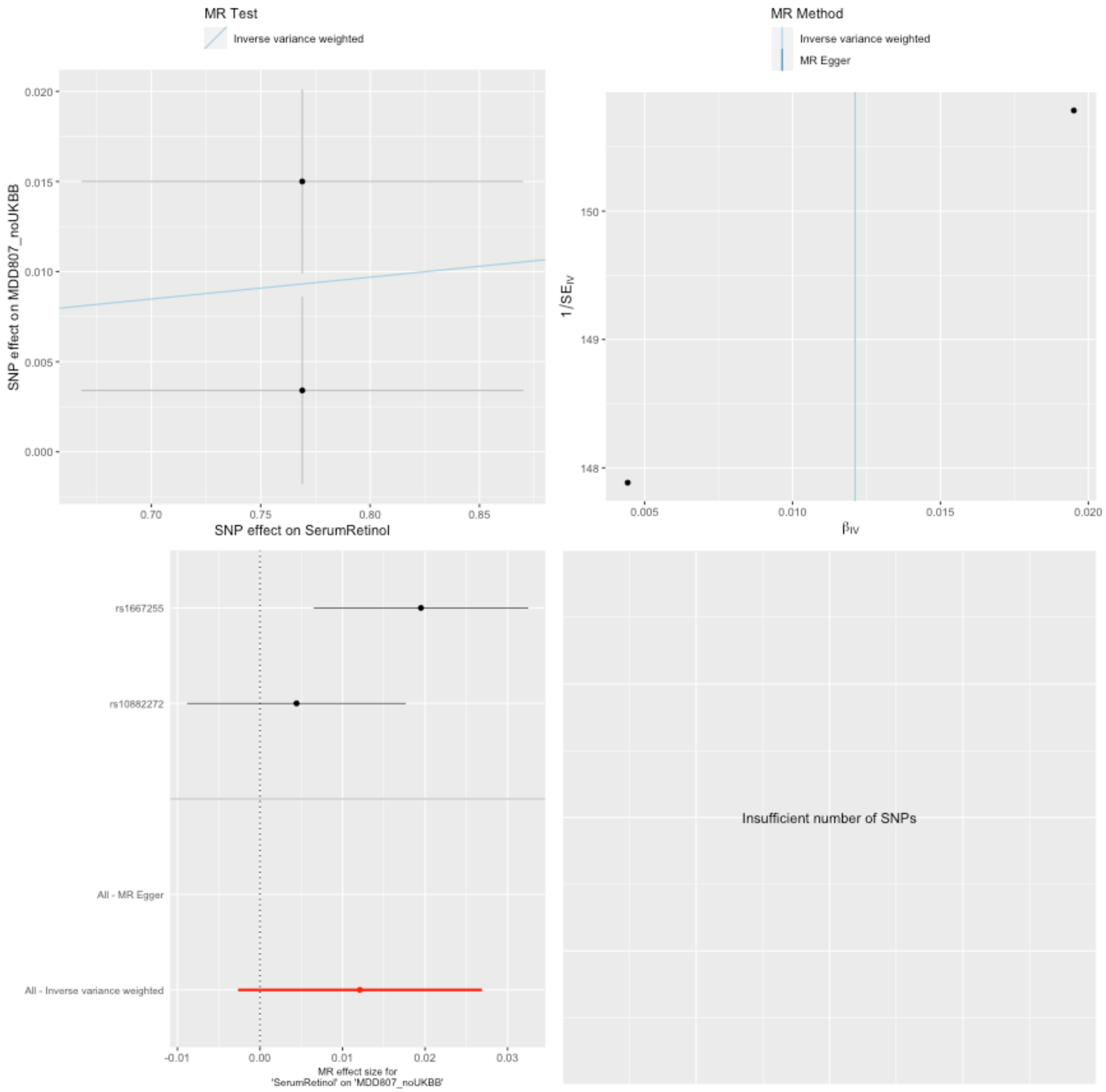

Serum Retinol and Recurrent Depression (N= 80,933)

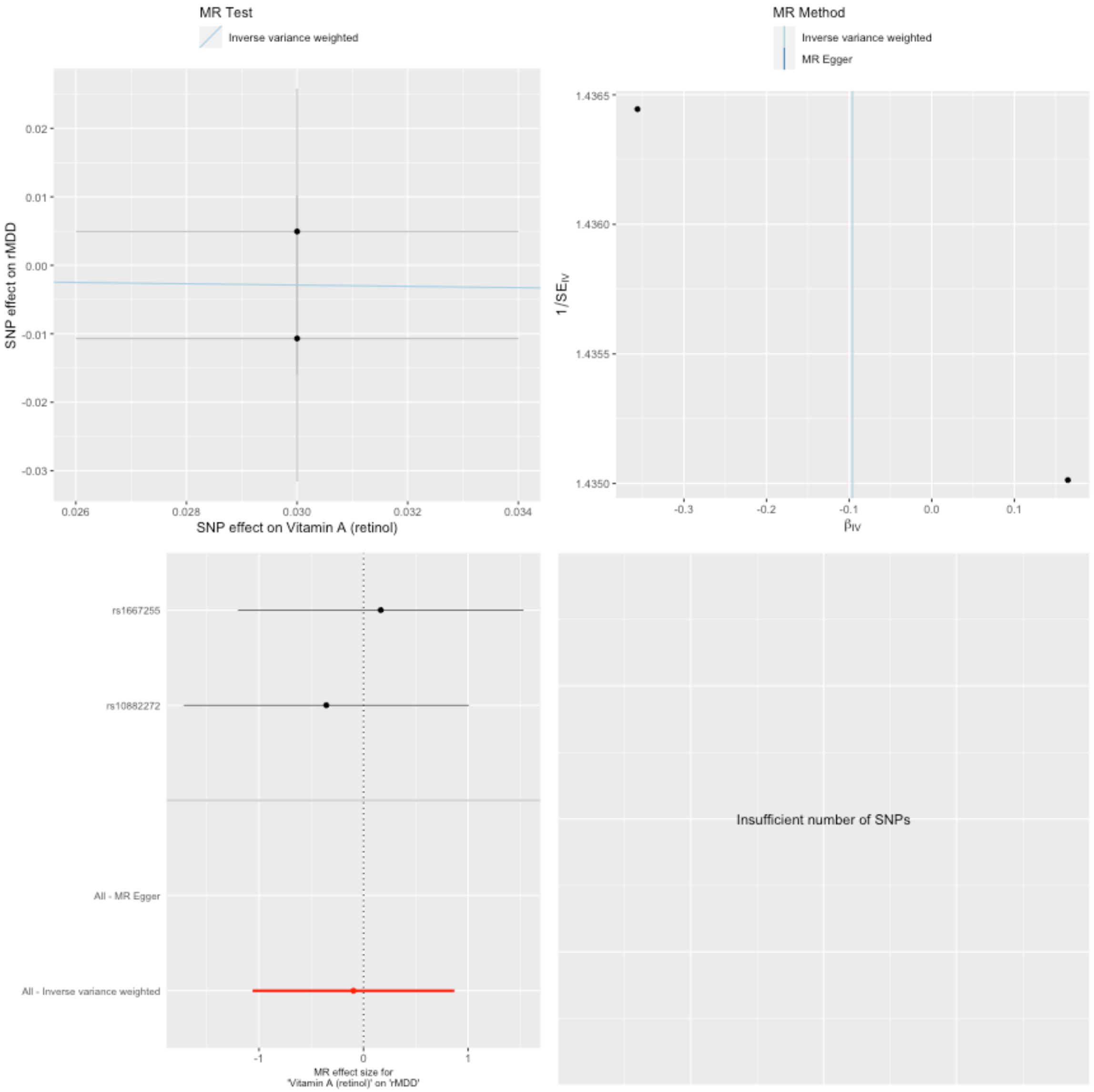

Serum 25 (OH) Vitamin D and Major Depressive Disorder (N=430,775)

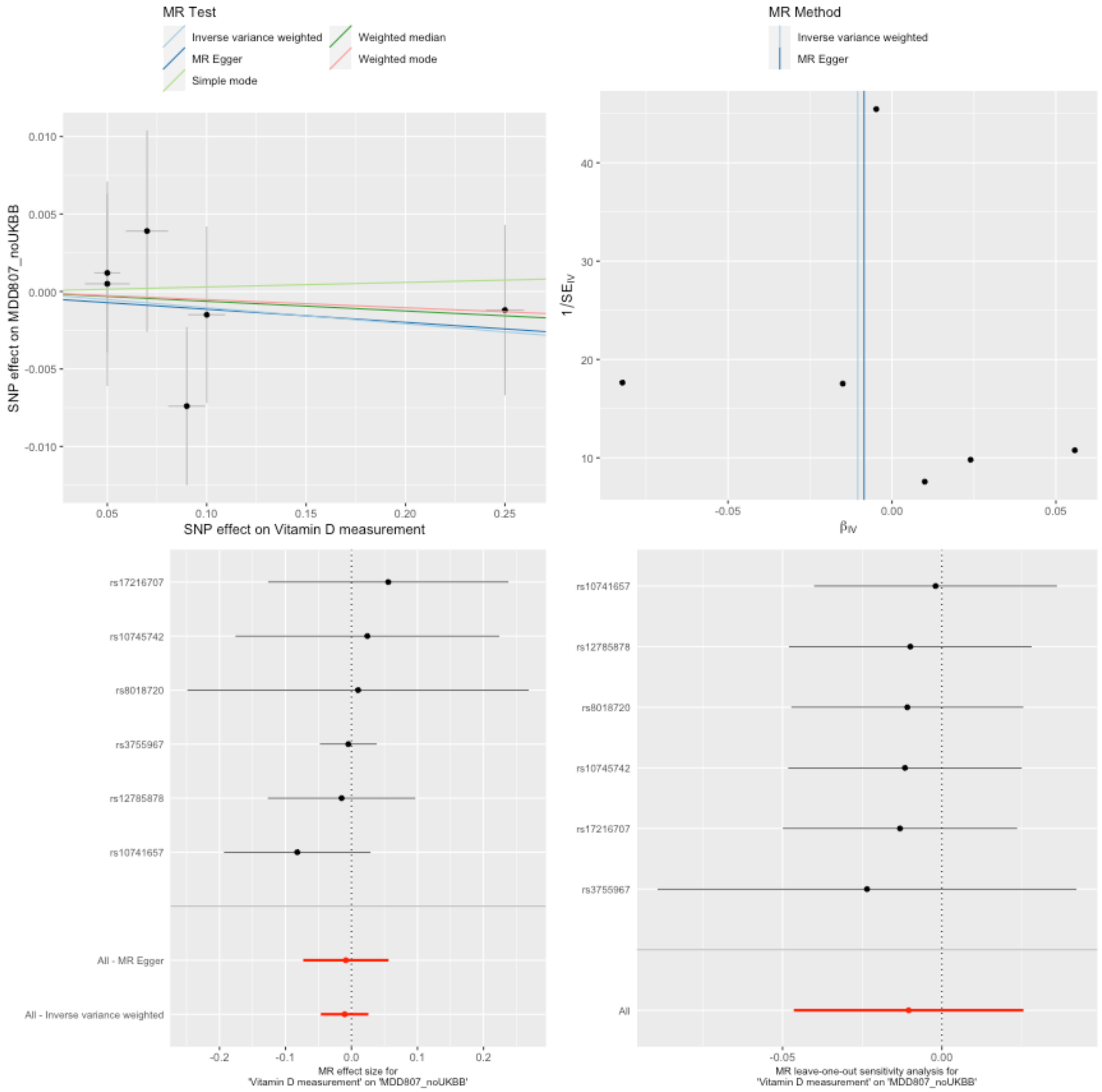

Macrominerals

Calcium and Major Depressive Disorder (N=430,775)

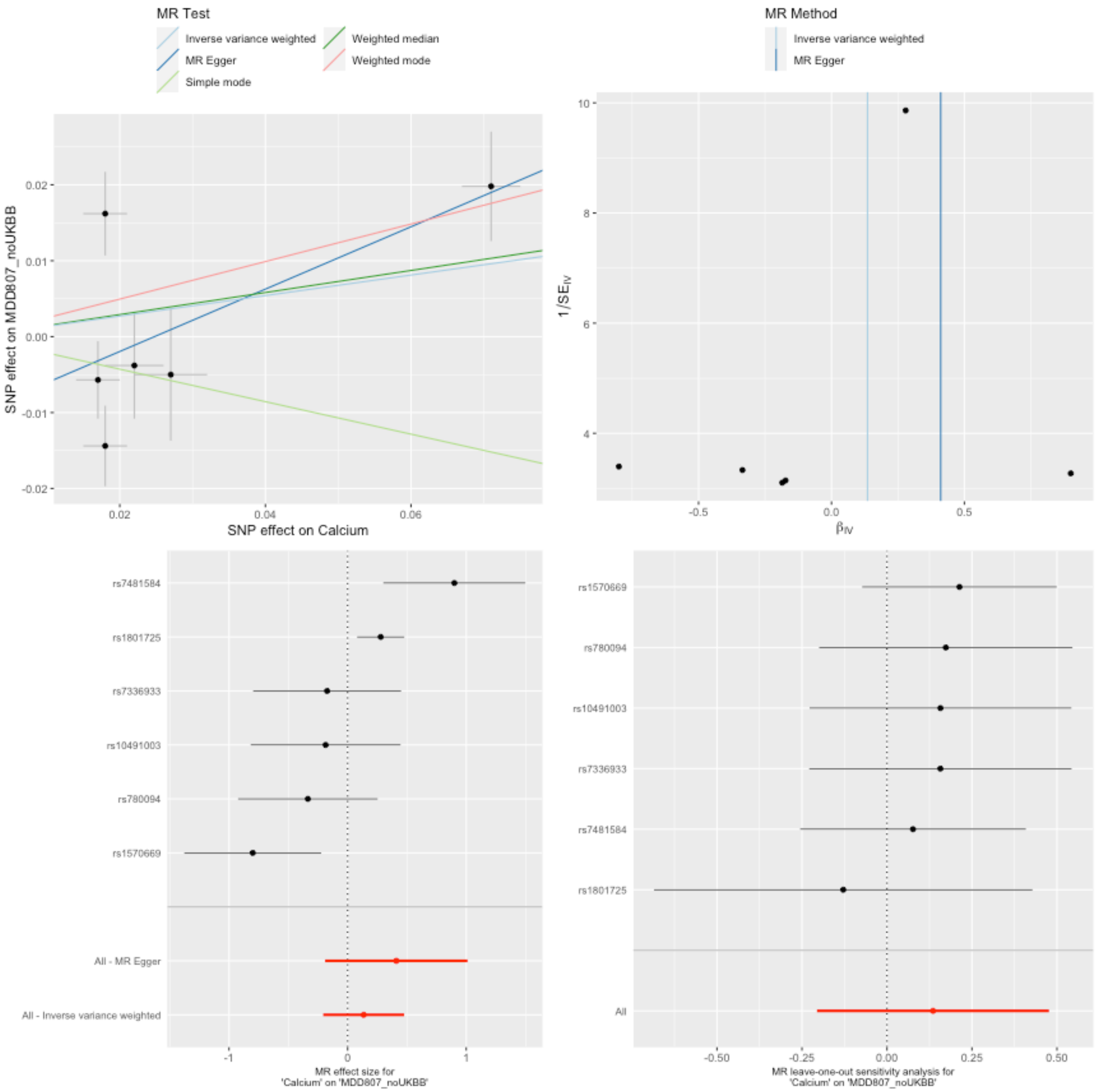

Calcium and Recurrent Depression (N= 80,933)

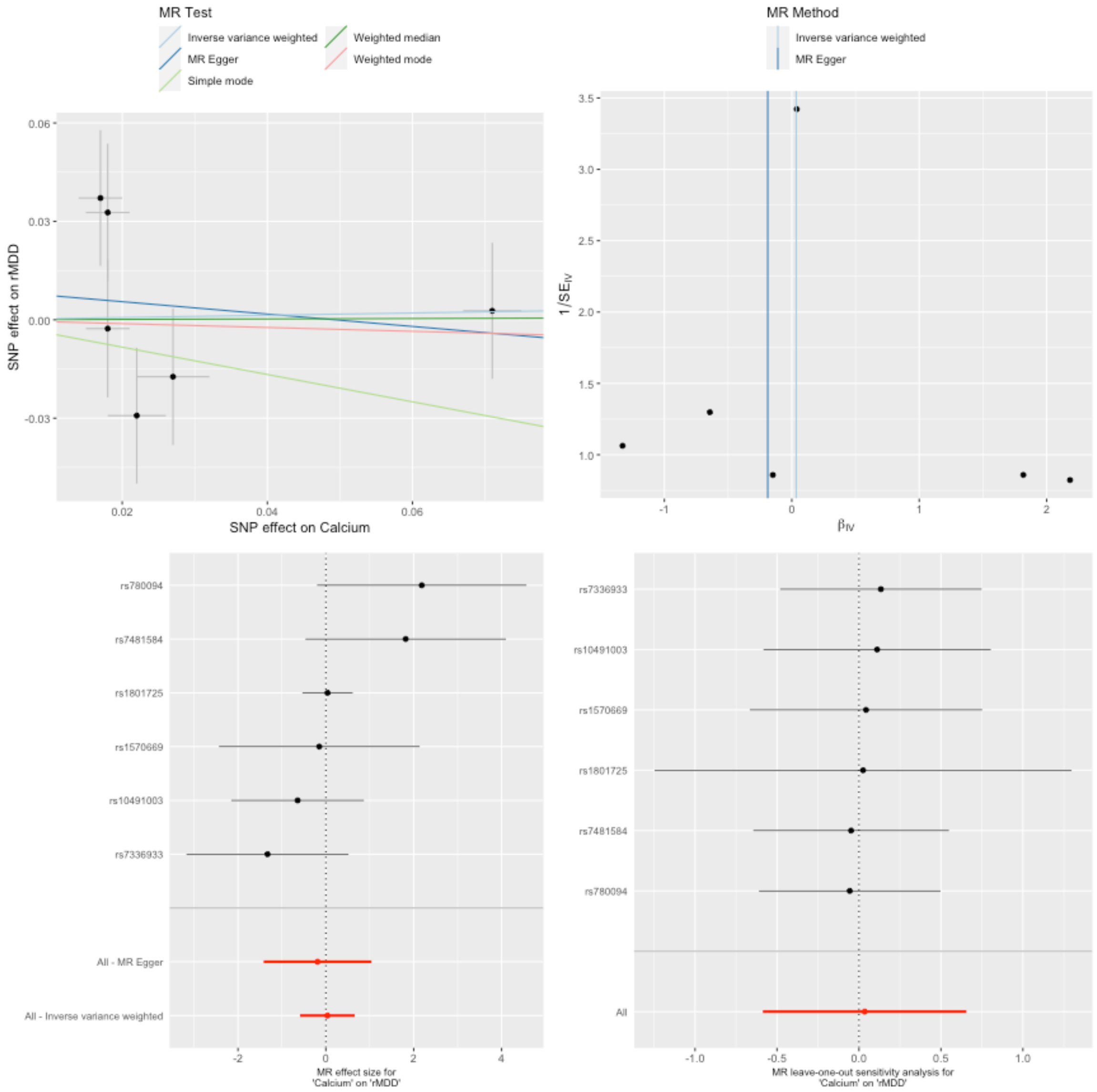

Serum Magnesium and Major Depressive Disorder (N=430,775)

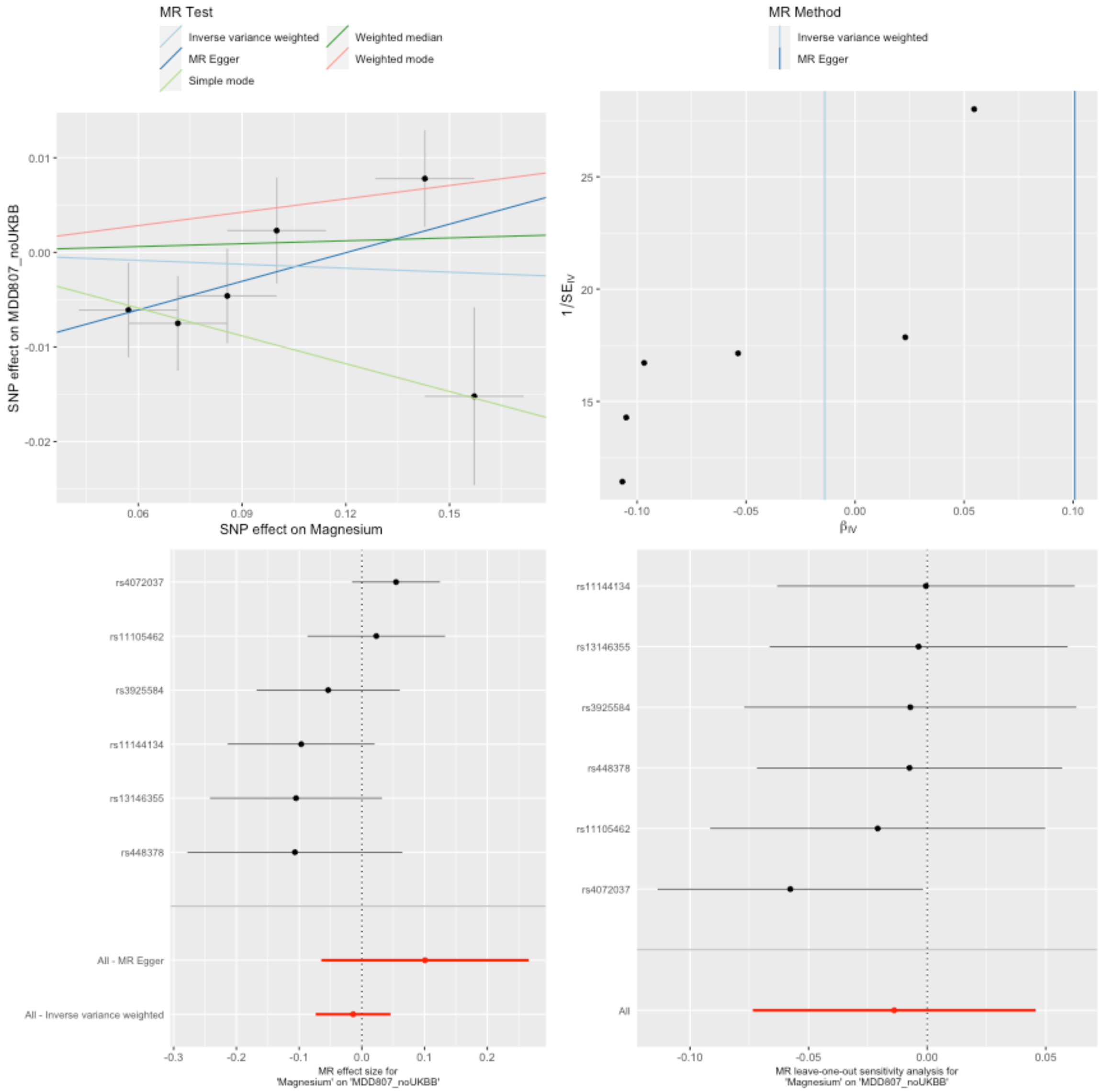

Serum Magnesium and Recurrent Depression (N= 80,933)

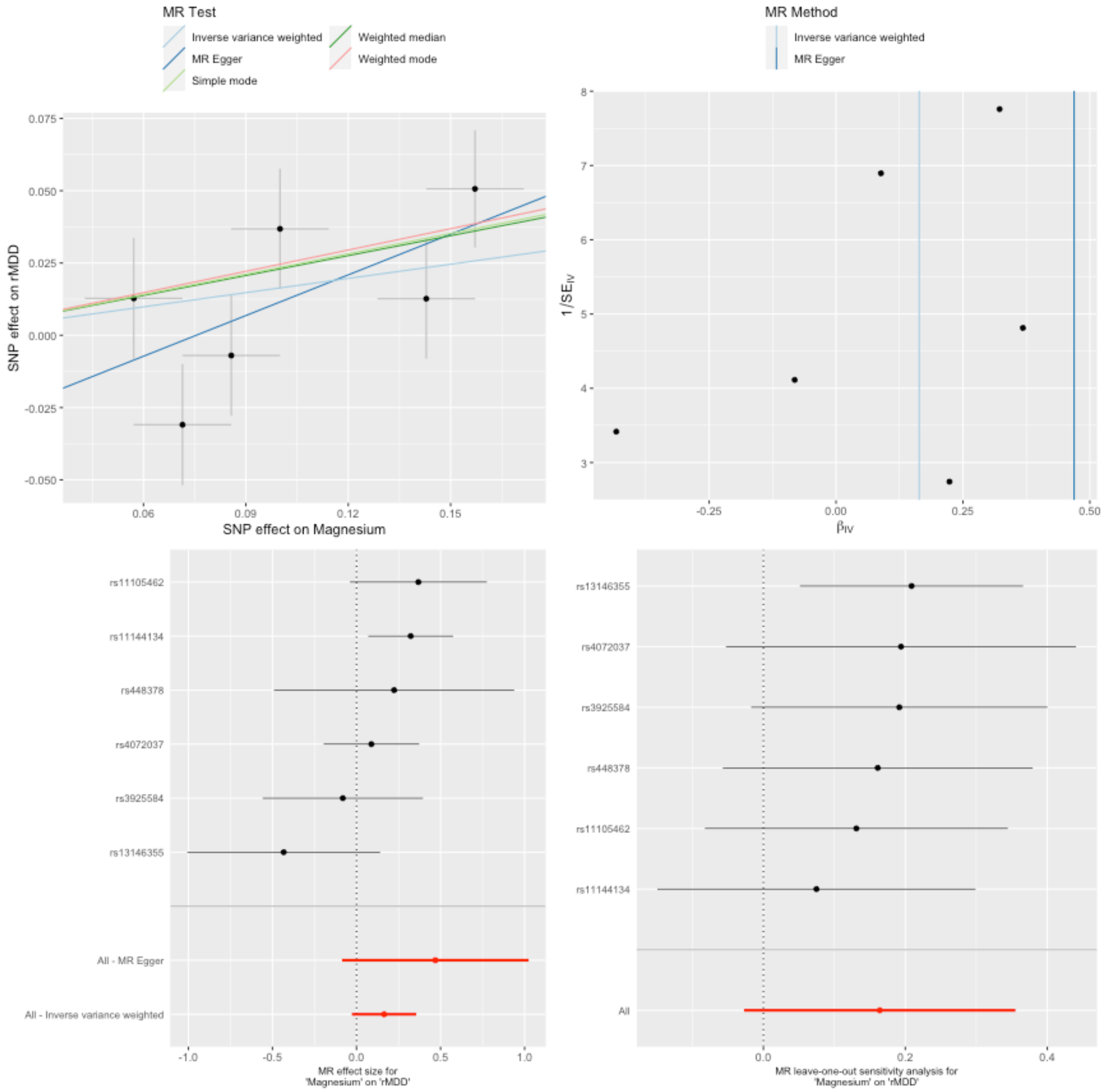

Phosphate and Major Depressive Disorder (N=430,775)

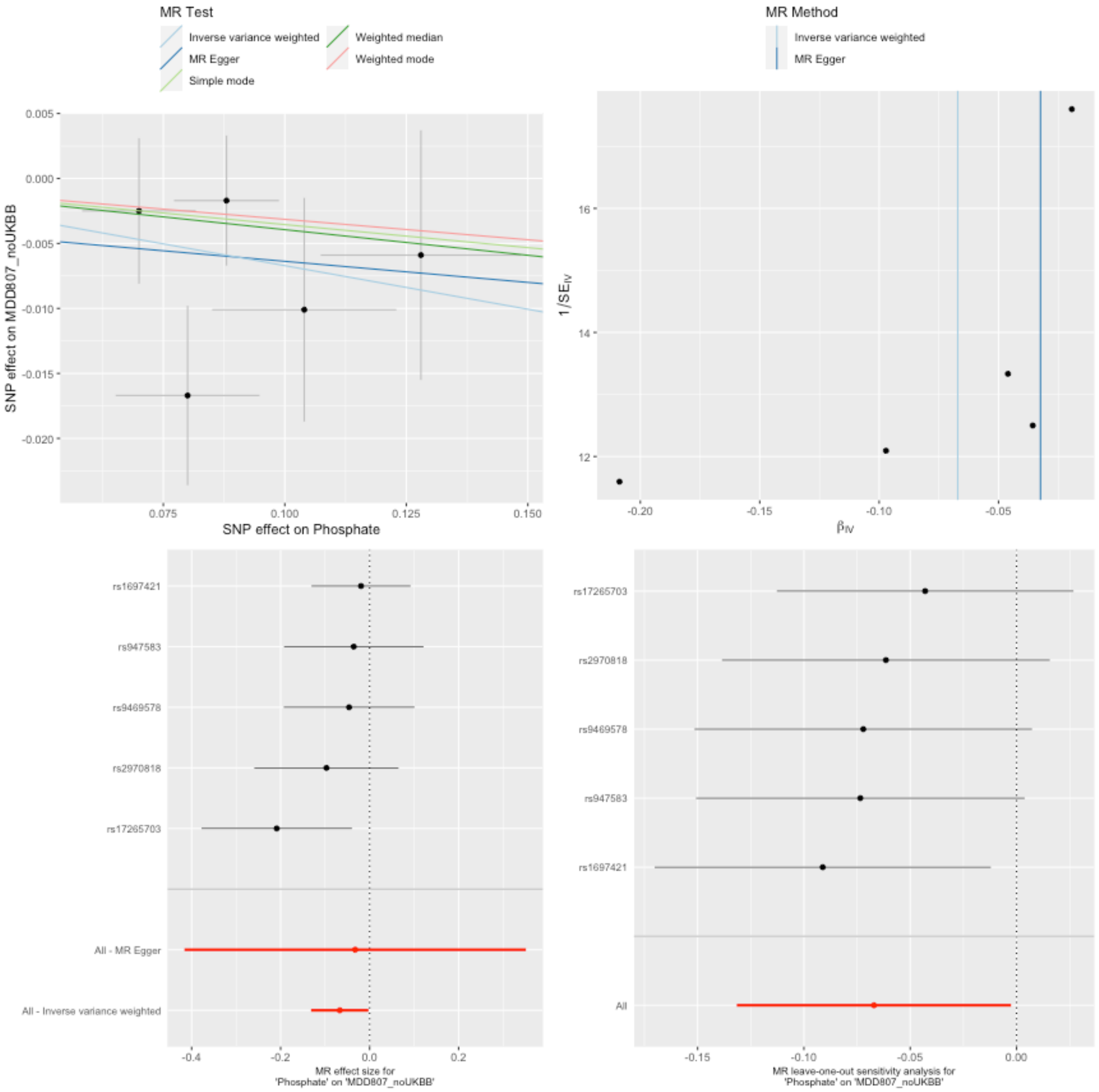

Phosphate and Recurrent Depression (N= 80,933)

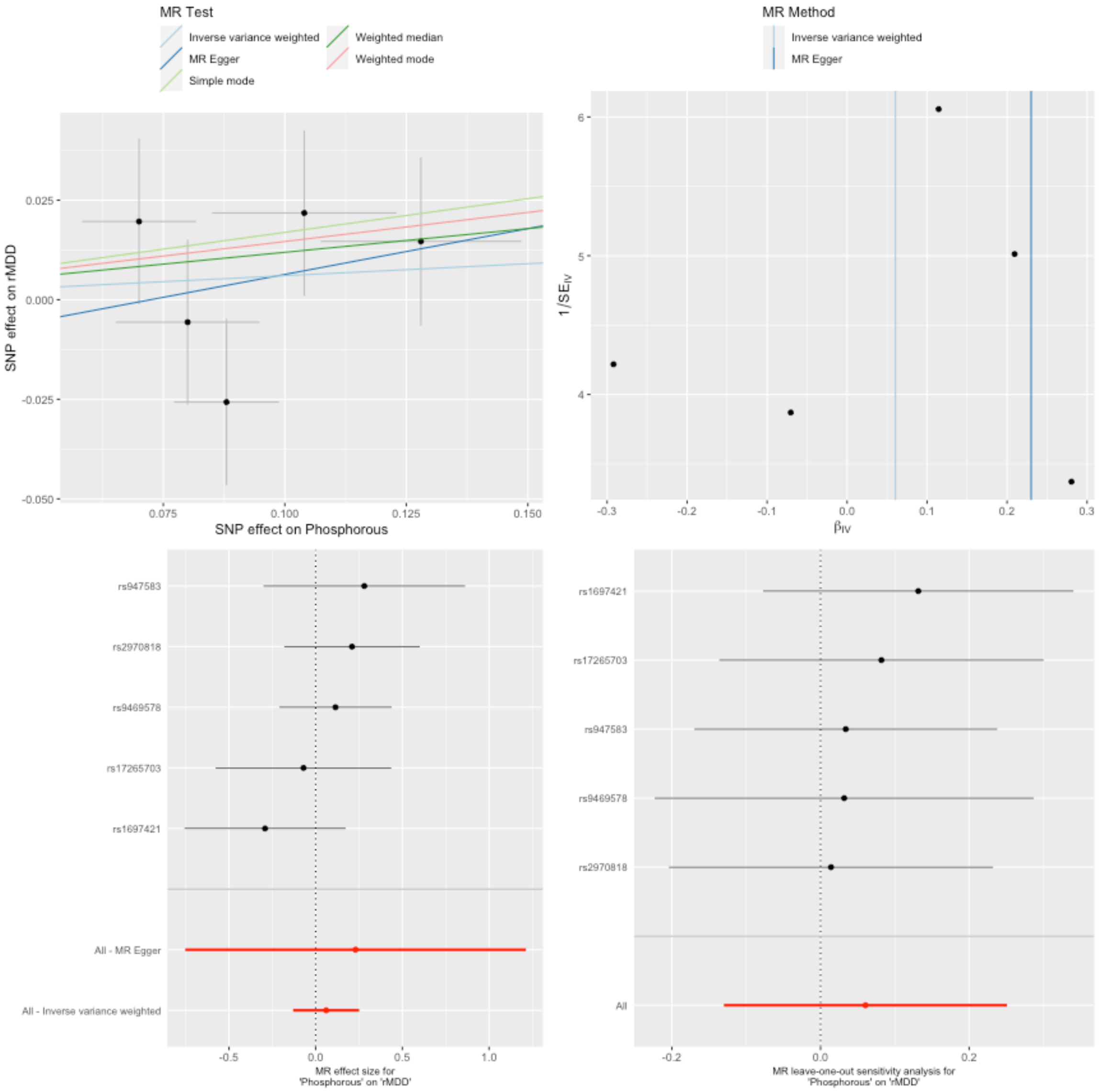

Microminerals

Serum Iron and Major Depressive Disorder (N=430,775)

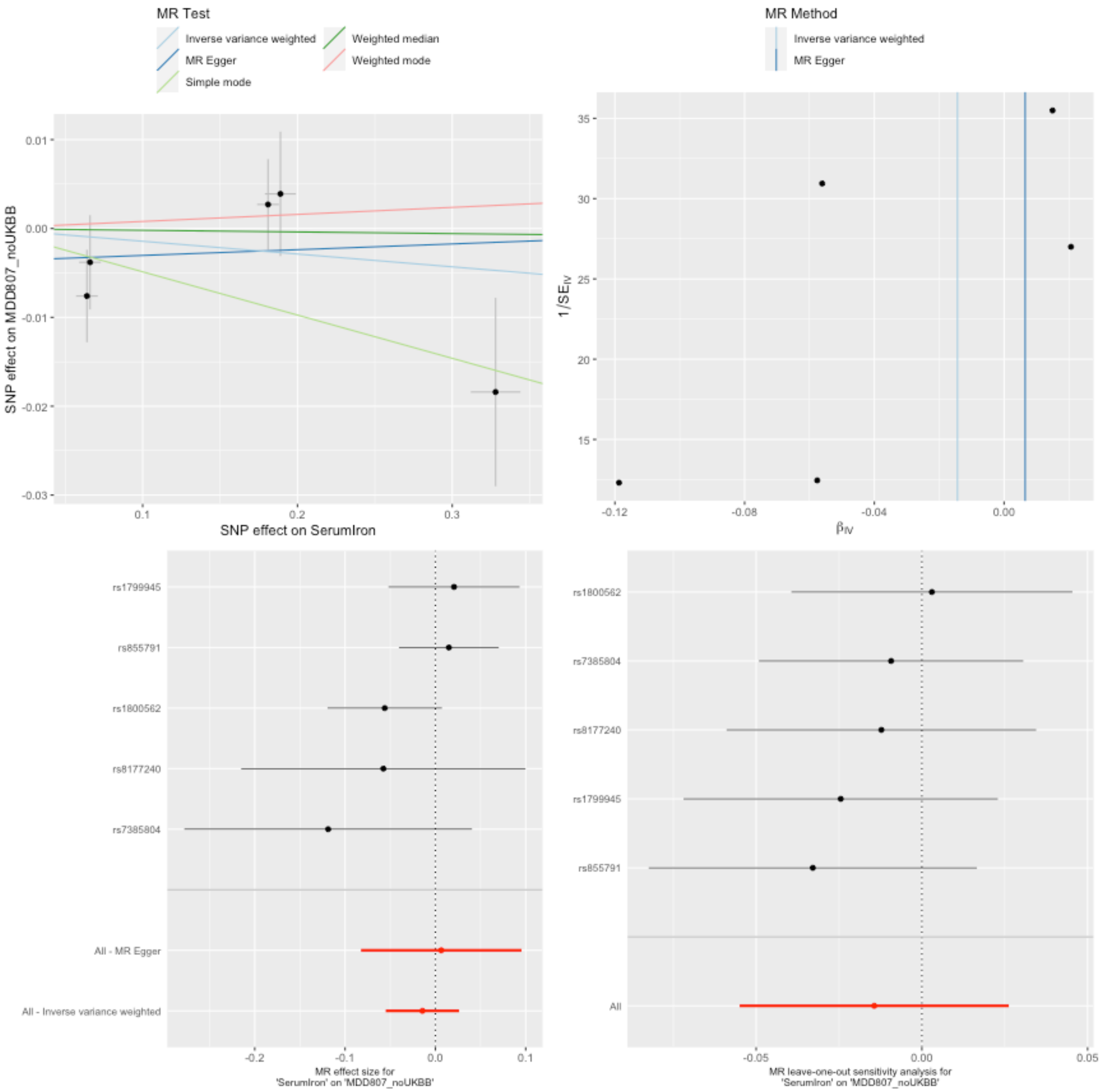

Serum Iron and Recurrent Depression (N= 80,933)

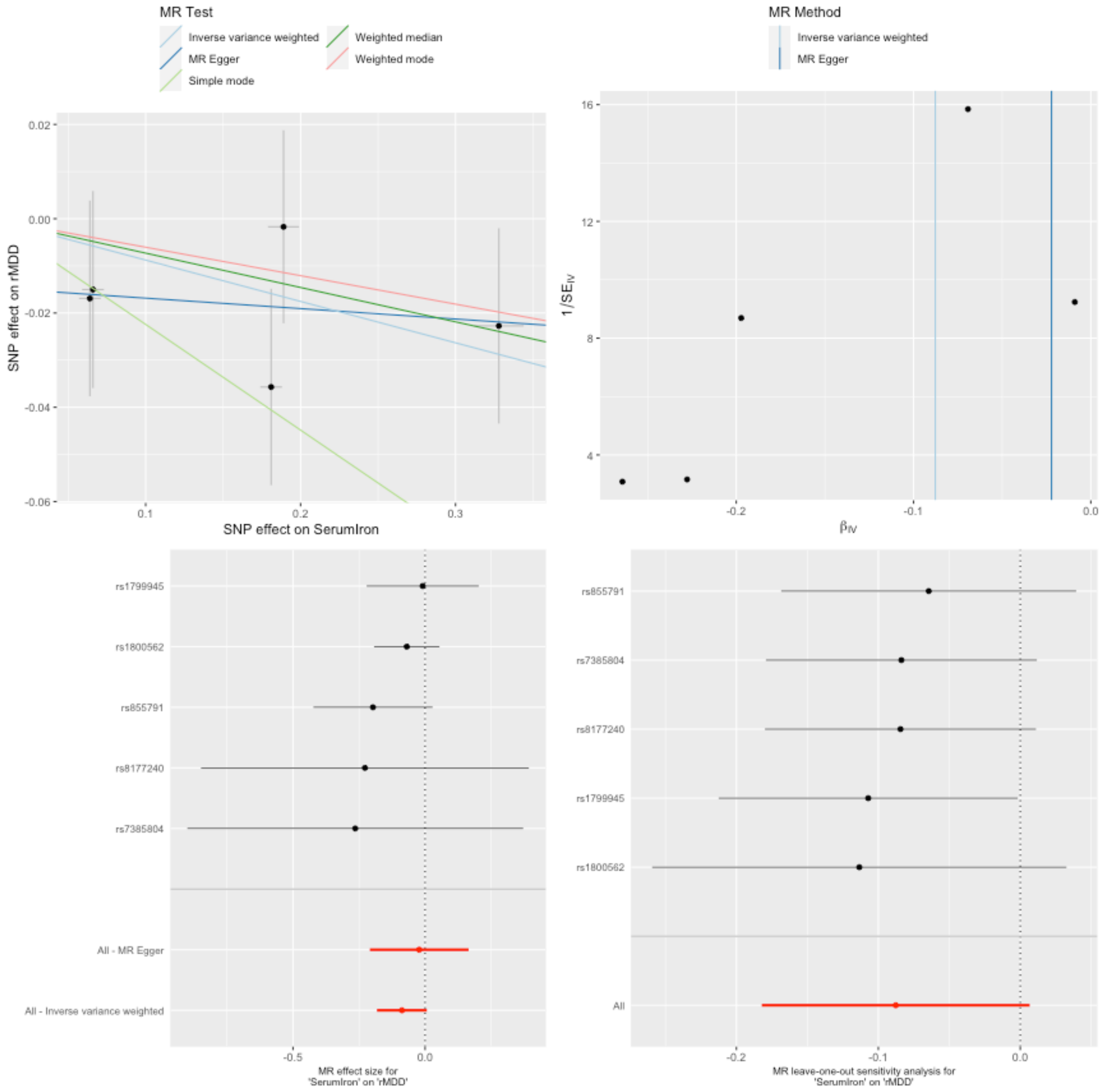

Serum Ferritin and Major Depressive Disorder (N=430,775)

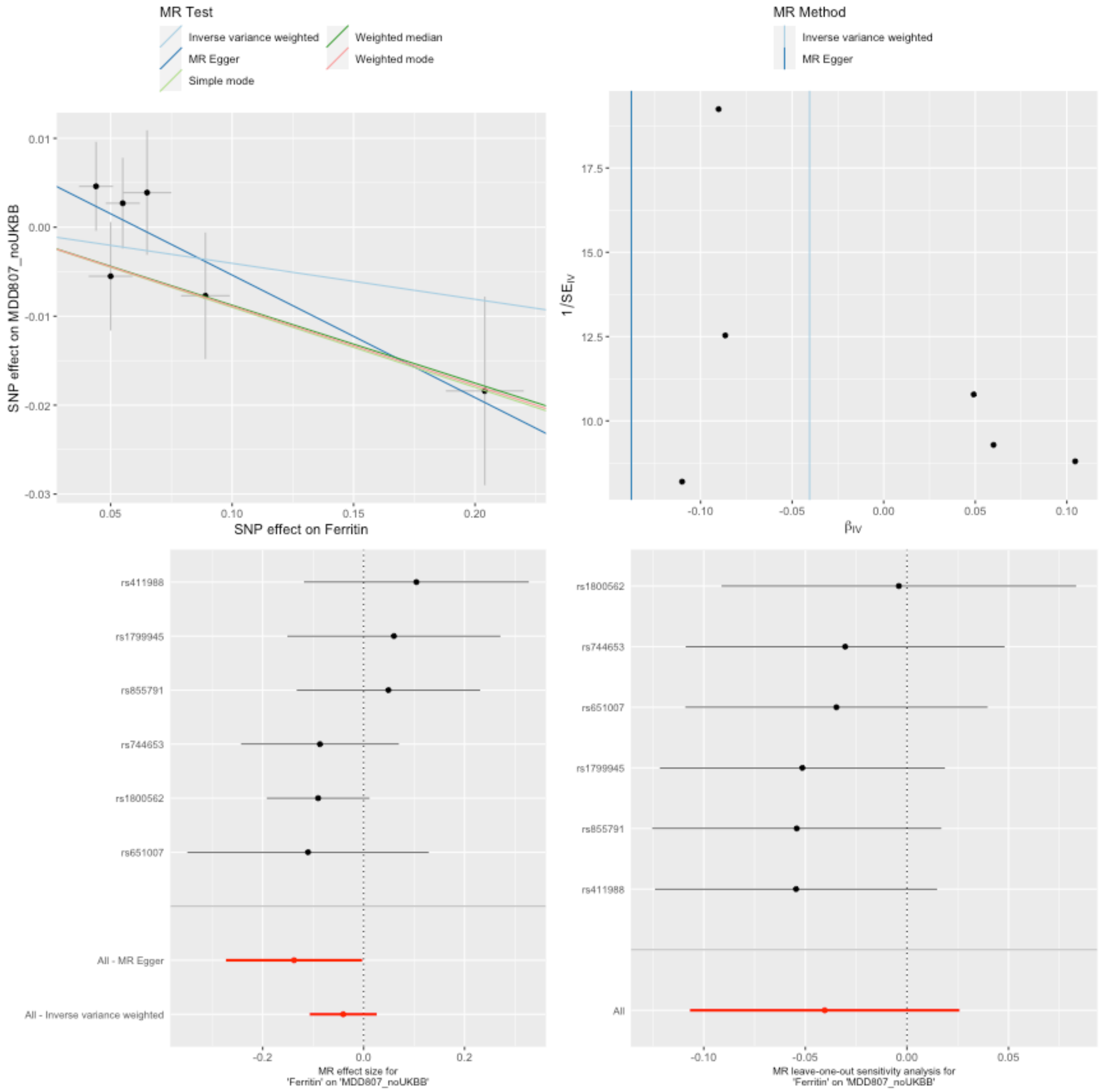

Serum Ferritin and Recurrent Depression (N= 80,933)

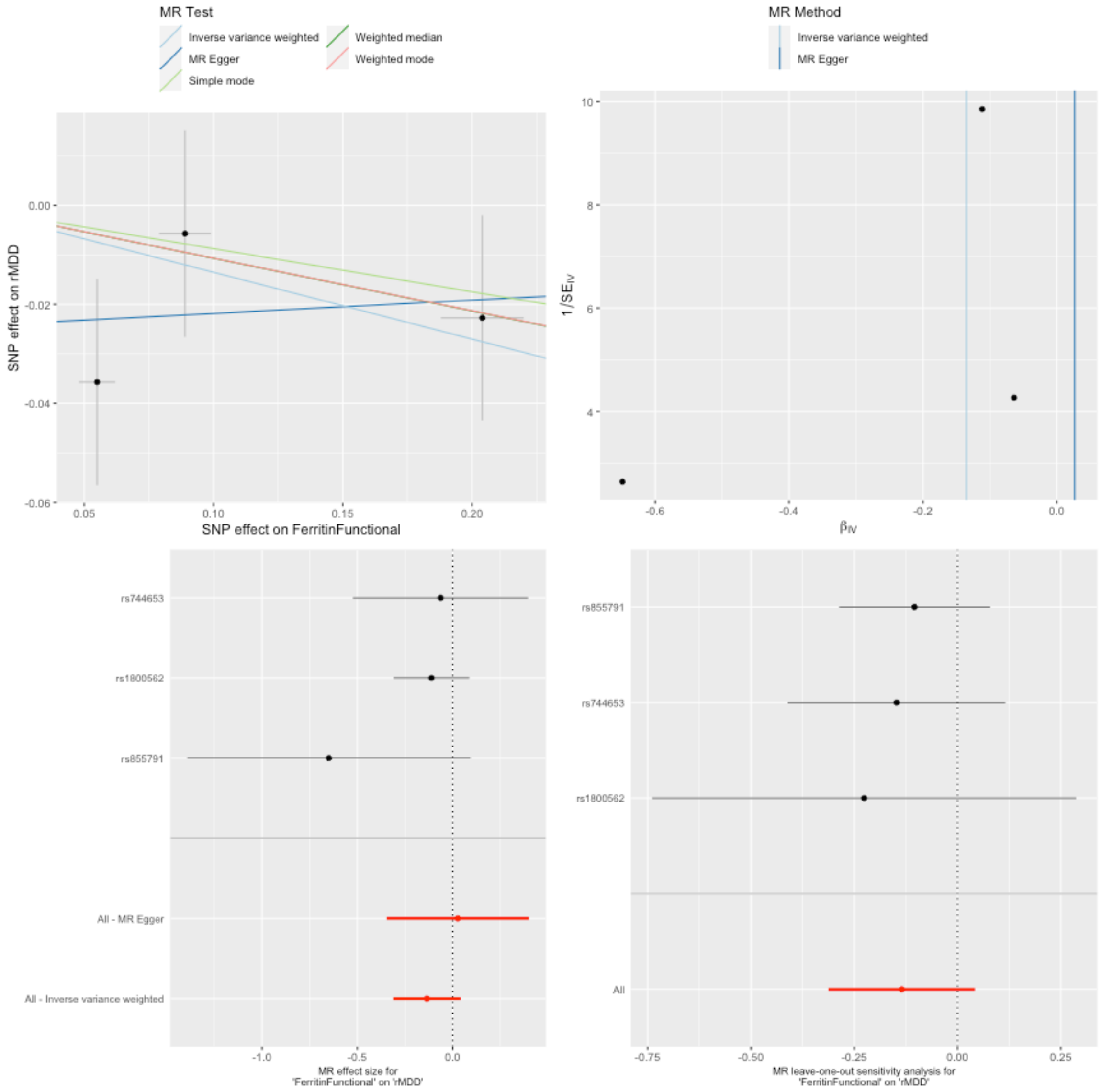

Erythrocyte Copper and Major Depressive Disorder (N=430,775)

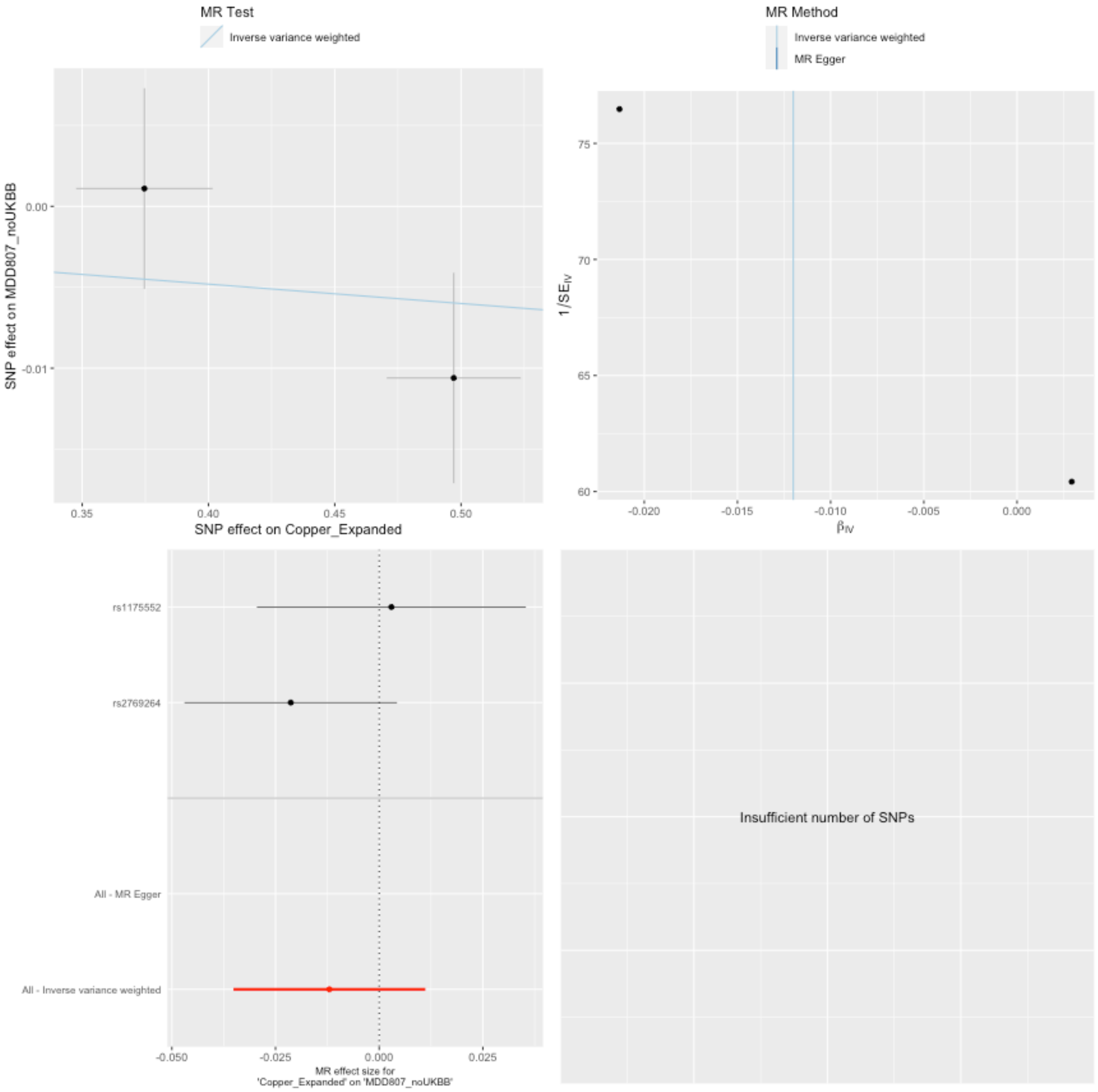

Erythrocyte Copper and Recurrent Depression (N= 80,933)

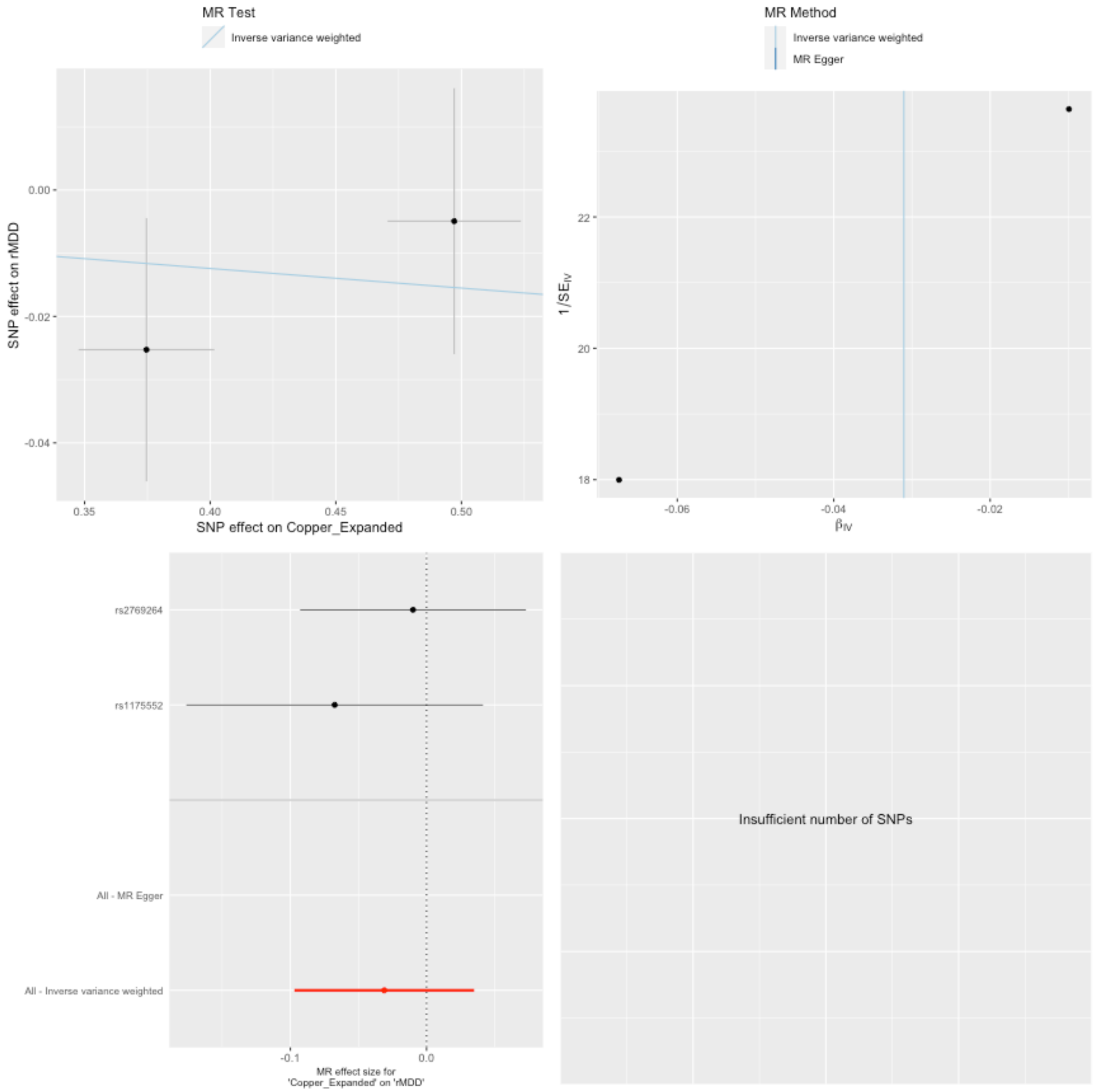

Serum Manganese and Major Depressive Disorder (N=430,775)

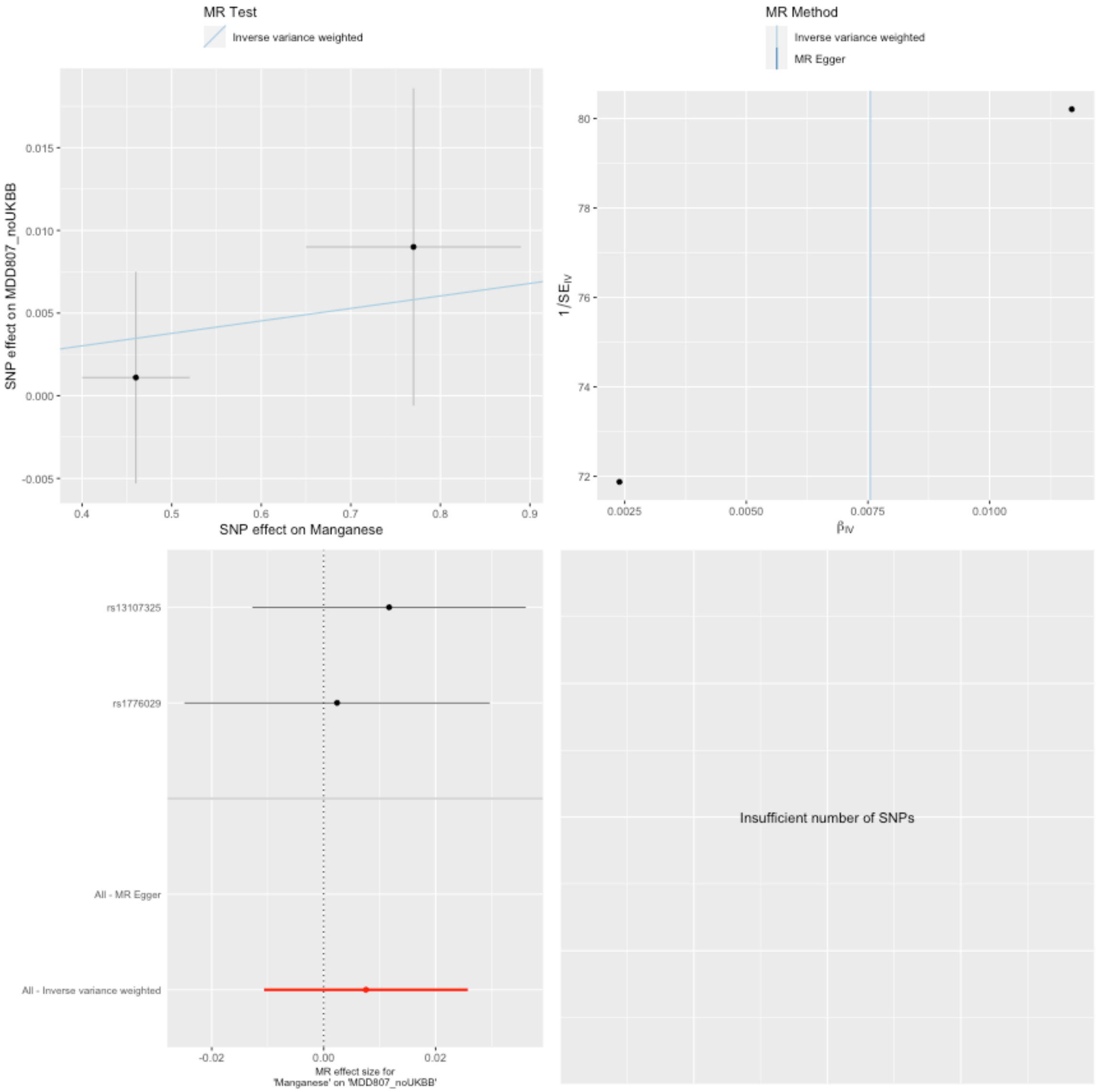

Serum Magnanese and Recurrent Depression (N= 80,933)

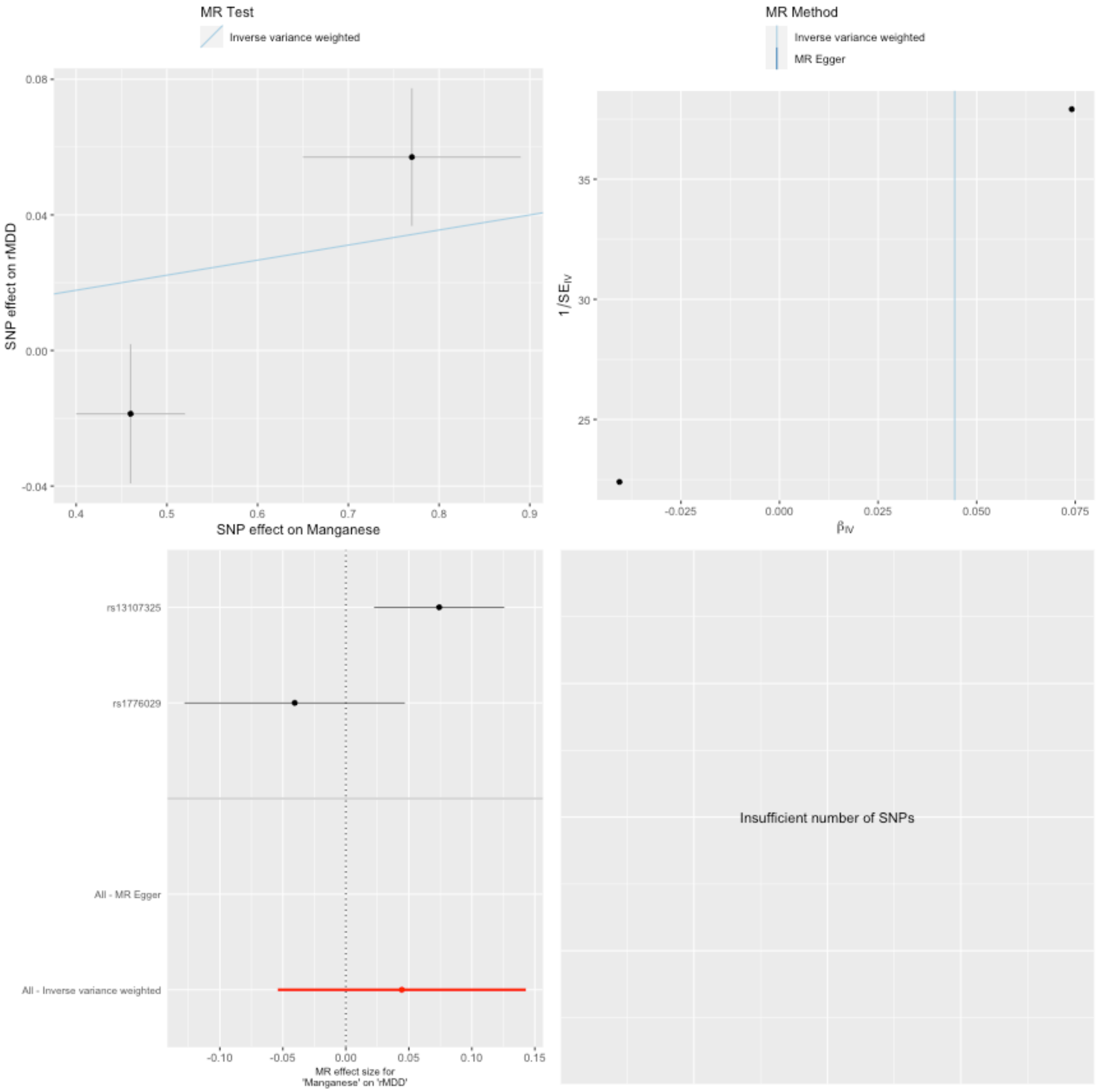

Serum Selenium and Major Depressive Disorder (N=430,775)

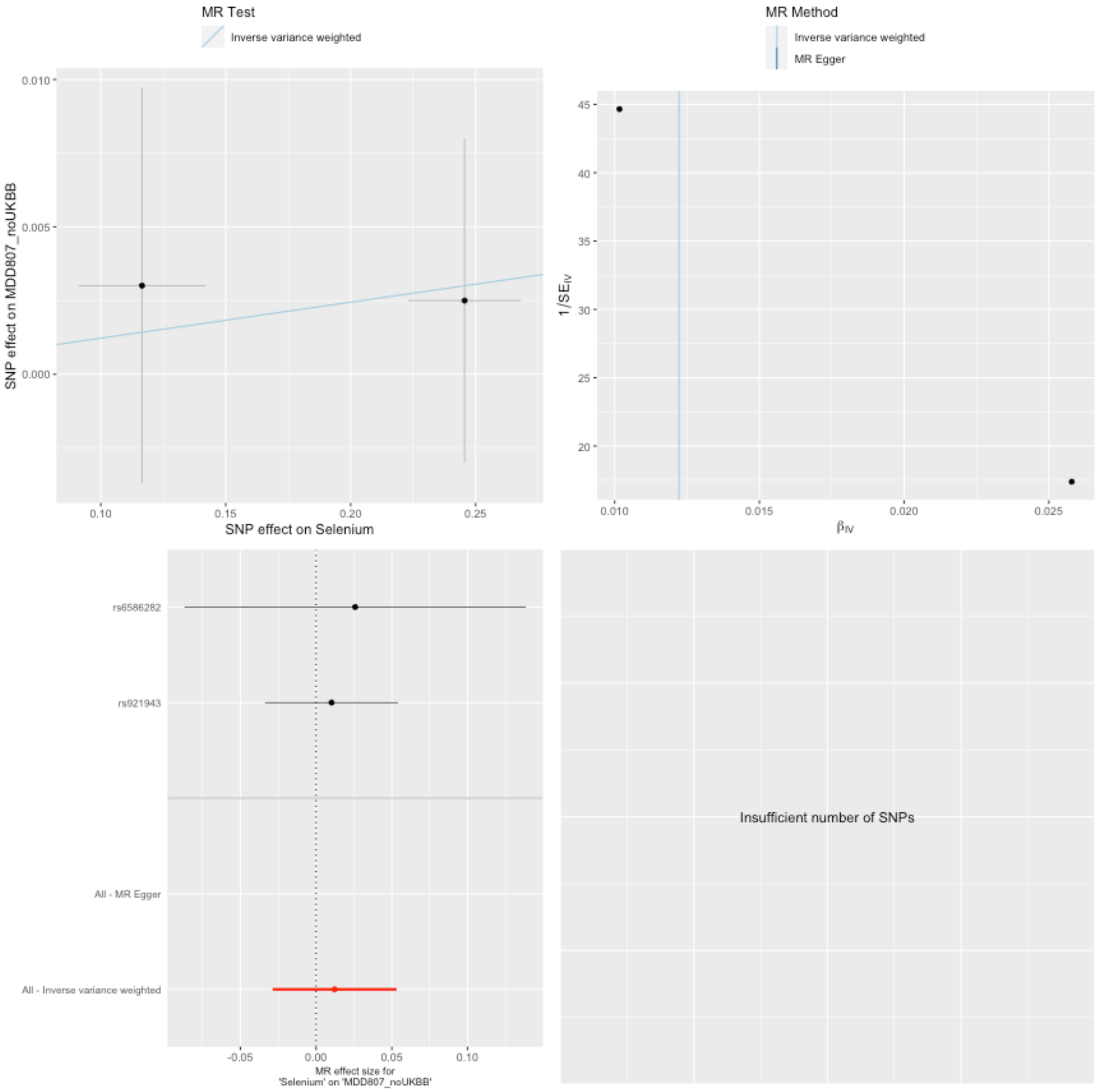

Serum Selenium and Recurrent Depression (N= 80,933)

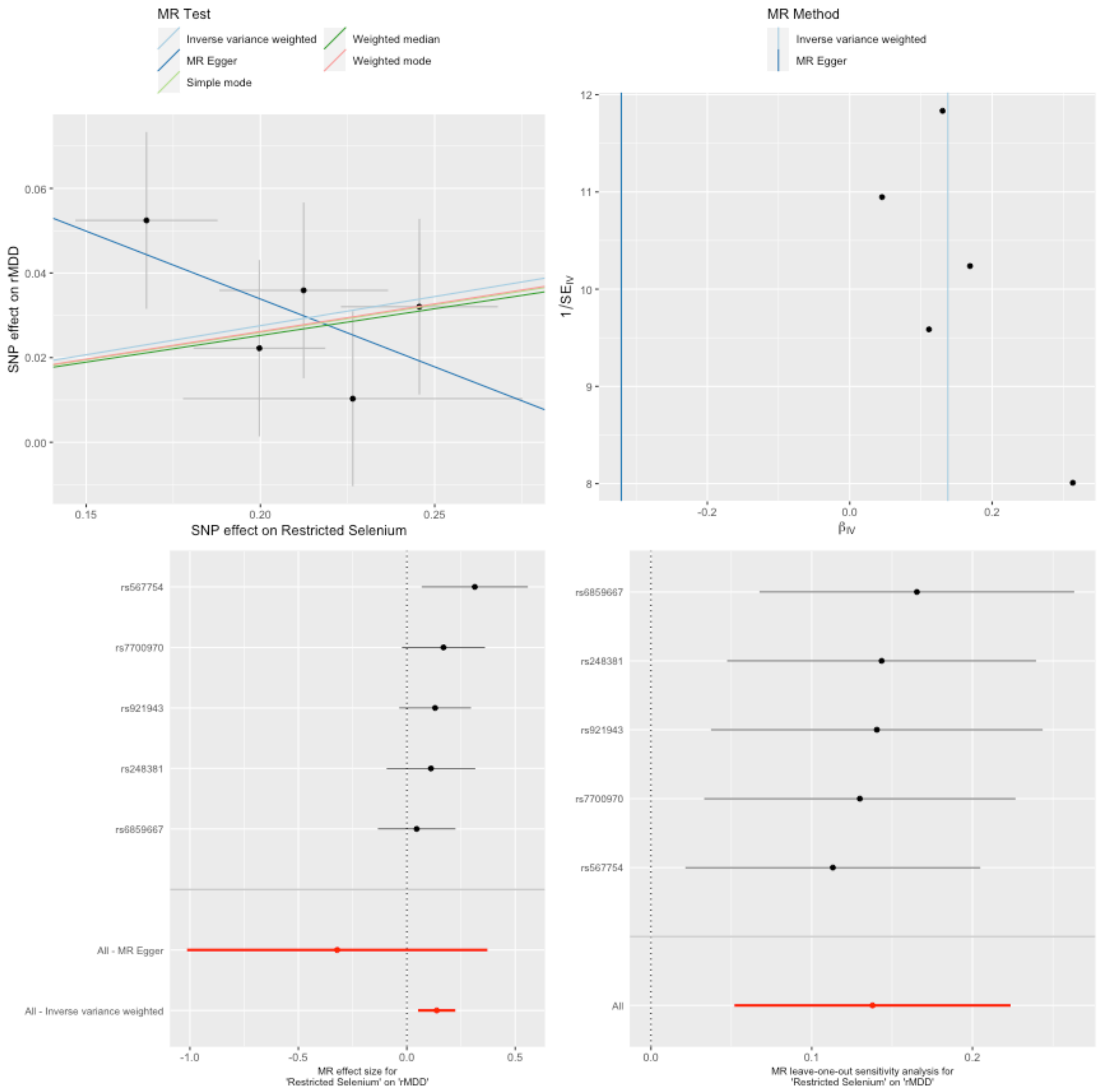

Erythrocyte Zinc and Major Depressive Disorder (N=430,775)

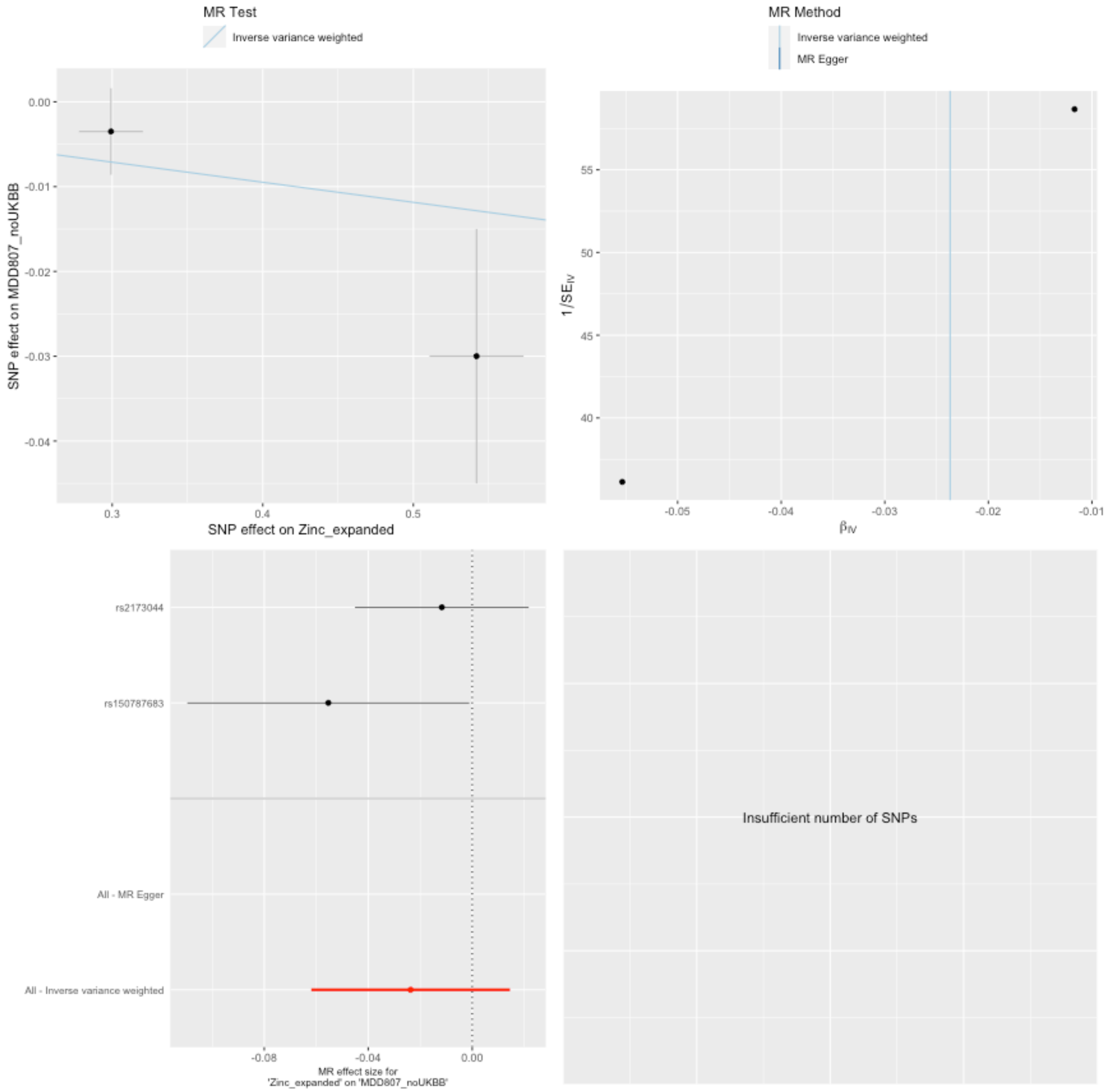

Erythrocyte Zinc and Recurrent Depression (N= 80,933)

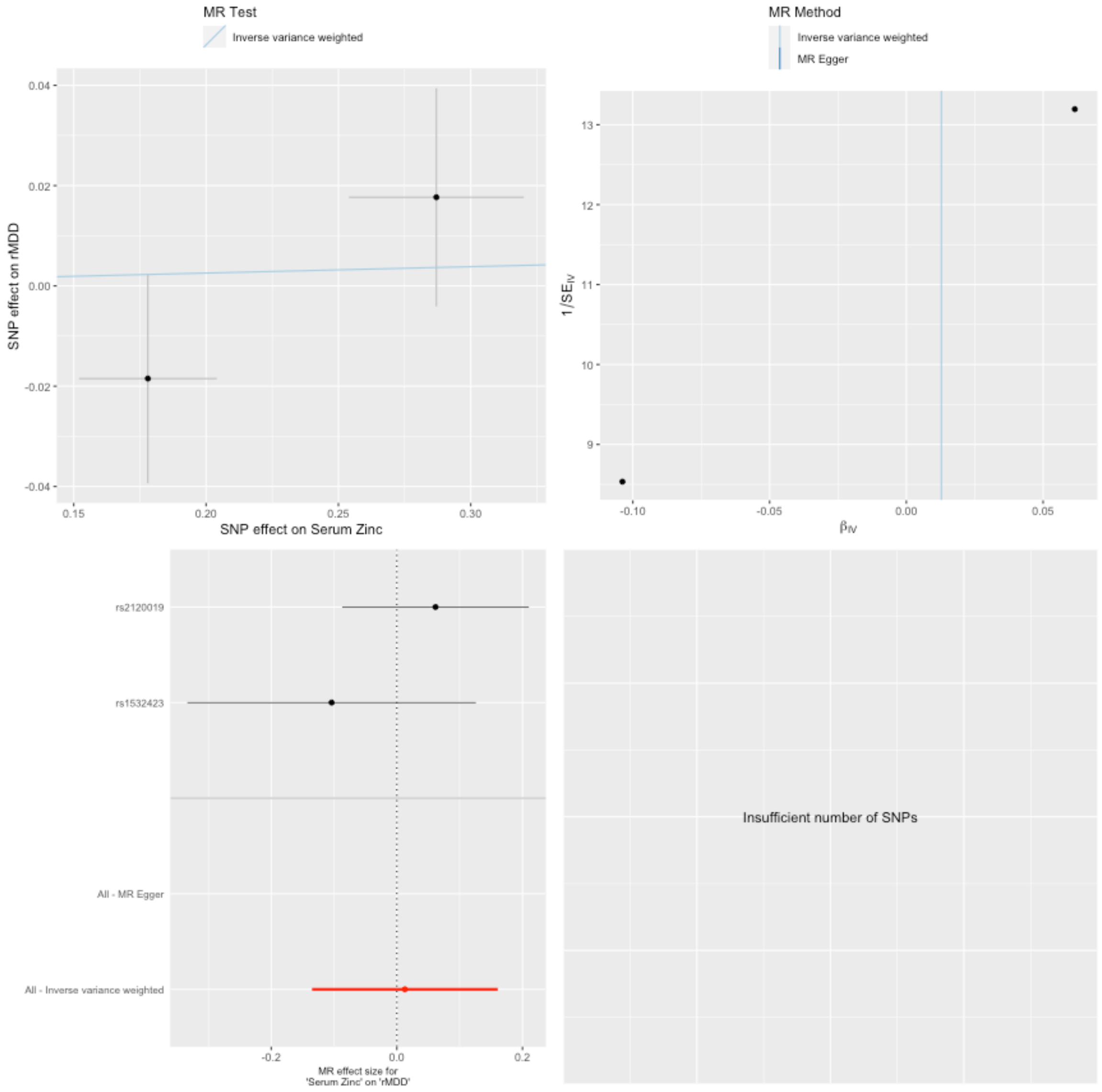

Supplementary Material S3: List of PGC MDD collaborators

Mark J Adams 1
Fabian Streit 2
Swapnil Awasthi 3
Brett N Adey 4
Karmel W Choi 5, 6
V Kartik Chundru 7
Jonathan RI Coleman 4, 8
Jerome C Foo 2
Olga Giannakopoulou 9
Alisha S M Hall 2, 10
Jens Hjerling-Leffler 11
David M Howard 4
Christopher Hübel 4, 12, 13
Alex S F Kwong 1, 14
Bochao Danae Lin 15
Xiangrui Meng 9
Guiyan Ni 16
Oliver Pain 17
Gita A Pathak 18, 19
Eva C Schulte 20, 21, 22, 23
Jackson G Thorp 24
Alicia Walker 16
Shuyang Yao 25
Jian Zeng 16
Johan Zvrskovec 4, 8
Dag Aarsland 26
Ky'Era V Actkins 27
Mazda Adli 3, 28
Esben Agerbo 12, 29, 30
Mareike Aichholzer 31
Tracy M Air 32
Allison Aiello 33
Thomas D Als 30, 34, 35
Evelyn Andersson 36
Till F M Andlauer 37, 38
Volker Arolt 39
Helga Ask 40, 41
Sunita Badola 42
Clive Ballard 43
Karina Banasik 44
Nicholas J Bass 9
Aartjan T F Beekman 45
Sintia Belangero 46
Elisabeth B Binder 38, 47
Ottar Bjerkeset 48, 49
Gyda Bjornsdottir 50
Julia Boberg 36
Sigrid Børte 51, 52, 53
Emma Bränn 54
Alice Braun 55
Thorsten Brodersen 56
Søren Brunak 44
Mie T Bruun 57
Pichit Buspavanich 58, 59
Jonas Bybjerg-Grauholm 60,61
Enda M Byrne 62
Archie Campbell 63, 64
Megan L. Campbell 65
Enrique Castelao 66
Jorge Cervilla 67, 68
Boris Chaumette 69
Chia-Yen Chen 70
Zhengming Chen 71, 72
Sven Cichon 73, 74, 75, 76
Lucía Colodro-Conde 24
Anne Corbett 43
Elizabeth C Corfield 40, 77
Baptiste Couvy-Duchesne 78
Nick Craddock 79, 80
Udo Dannlowski 39
Gail Davies 81
EJC de Geus 82
Ian J Deary 81
Franziska Degenhardt 76, 83
Abbas Dehghan 84, 85
J Raymond DePaulo 86
Michael Deuschle 87
Maria Didriksen 88
Khoa Manh Dinh 89
Nese Direk 90
Srdjan Djurovic 91, 92
Anna R Docherty 93, 94, 95
Katharina Domschke 96
Joseph Dowsett 88
Ole Kristian Drange 49, 97,98, 99
Erin C Dunn 6, 100
Gudmundur Einarsson 50
Thalia C Eley 4
Samar S M Elsheikh 101
Jan Engelmann 102
Michael E Benros 60, 103,104
Christian Erikstrup 89
Valentina Escott-Price 80
Chiara Fabbri 4, 105
Yu Fang 106
Sarah Finer 107
Josef Frank 2
Robert C Free 108
He Gao 109

Michael Gill 110
Maria Gilles 87
Fernando S Goes 86
Scott Douglas Gordon 24
Jakob Grove 30, 34, 35, 111
Daniel F Gudbjartsson 50,112
Blanca Gutierrez 67, 68
Tim Hahn 39
Lynsey S Hall 80
Thomas F Hansen 44, 60,113
Magnus Haraldsson 114
Catherina A Hartman 115
Alexandra Havdahl 40
Caroline Hayward 116
Stefanie Heilmann-Heimbach 76
Stefan Herms 74, 76
Ian B Hickie 117
Henrik Hjalgrim 118
Per Hoffmann 74, 76
Georg Homuth 119
Carsten Horn 120
Jouke-Jan Hottenga 82
David M Hougaard 60, 61
Iiris Hovatta 121
Qin Qin Huang 7
Floris Huider 82
Karen A Hunt 122
Marcus Ising 123
Erkki Isometsä 124
Rick Jansen 45
Yunxuan Jiang 125
Ian Jones 80
Lisa A Jones 126
Lina Jonsson 127
Robert Karlsson 25
Siegfried Kasper 128
Kenneth S Kendler 129
Ronald C Kessler 130
Stefan Kloiber 101, 123, 131,132
James A Knowles 133
Nastassja Koen 65-22-
Julia Kraft 55
Henry R Kranzler 134, 135
Kristi Krebs 136
Theodora Kunovac Kallak137
Zoltán Kutalik 138, 139, 140
Elisa Lahtela 141
Margit Hørup Larsen 88
Eric J Lenze 142
Daniel F Levey 143, 144
Melissa Lewins 1
Glyn Lewis 9
Liming Li 145, 146
Kuang Lin 71
Penelope A Lind 24
Donald J MacIntyre 1, 147,148
Dean F MacKinnon 86
Hermine HM Maes 149, 150
Wolfgang Maier 151
Victoria S Marshe 101, 152
Hamdi Mbarek 82
Peter McGuffin 4
Sarah E Medland 24
Susanne Meinert 39, 153
Susan Mikkelsen 89
Christina Mikkelsen 88, 154
Yuri Milaneschi 45
Iona Y Millwood 71, 72
Brittany L Mitchell 24
Esther Molina 67, 155
Francis M Mondimore 86
Preben Bo Mortensen 12,29, 30
Benoit H Mulsant 101, 131
Joonas Naamanka 121
Jake M Najman 156
Matthias Nauck 157, 158
Igor Nenadić 159
Kasper R Nielsen 160
Ilja M Nolte 161
Merete Nordentoft 60, 103,104
Markus M Nöthen 76
Mette Nyegaard 30, 162,163, 164
Michael C O'Donovan 80
Asmundur Oddsson 50
Catherine M Olsen 165, 166
Hogni Oskarsson 167
Sisse Rye Ostrowski 88, 168
Vanessa K Ota 46
Michael J Owen 80
Richard Packer 169
Teemu Palviainen 141
Pedro M Pan 170
Carlos N Pato 171
Michele T Pato 171
Nancy L Pedersen 25
Ole Birger Pedersen 172
Roseann E Peterson 129,173
Wouter J Peyrot 45
James B Potash 86
Martin Preisig 66
Jorge A Quiroz 174

Charles F Reynolds III 175
John P Rice 142
Giovanni A Salum 176
Robert A Schoevers 177, 178
Andrew Schork 30, 179, 180
Thomas G Schulze 2, 21, 86,181, 182
Tabea S Send 87
Jianxin Shi 183
Engilbert Sigurdsson 114
Kritika Singh 27
Grant C B Sinnamon 184
Lea Sirignano 2
Olav B Smeland 185, 186
Daniel J Smith 187
Erik Sørensen 88
Sundararajan Srinivasan 188
Hreinn Stefansson 50
Kari Stefansson 50, 189
Dan J. Stein 190
Frederike Stein 191
André Tadic 102, 192
Henning Teismann 193
Alexander Teumer 194
Anita Thapar 80, 195
Pippa A Thomson 64
Lise Wegner Thørner 88
Apostolia Topaloudi 196
Ioanna Tzoulaki 84, 85, 197
Monica Uddin 198
André G Uitterlinden 199
Henrik Ullum 88, 200, 201
Daniel Umbricht 202
Robert J Ursano 203
Sandra Van der Auwera 204
David A van Heel 122
Albert M van Hemert 205
Abirami Veluchamy 188
Alexander Viktorin 25
Henry Völzke 194
Agaz Wani 198
G Bragi Walters 50
Robin G Walters 71, 72
Sylvia Wassertheil-Smoller206
Myrna M Weissman 207,208
Jürgen Wellmann 193
David C Whiteman 165
Derek Wildman 198
Gonneke Willemsen 82
Alexander T Williams 169
Bendik S Winsvold 51, 52,209
Stephanie H Witt 2
Ying Xiong 25
Lea Zillich 2
John-Anker Zwart 51, 52, 53
23andMe Research Team125
Estonian Biobank Research
Team 136
HUNT All-In Psychiatry 210
China Kadoorie Biobank
Collaborative Group 211
Genes & Health Research
Team 212
Ole A Andreassen 185, 186,213
Bernhard T Baune 214, 215,216
Klaus Berger 193
Dorret I Boomsma 82
Anders D Børglum 30, 34, 35
Gerome Breen 4, 8
Na Cai 217, 218, 219
Hilary Coon 94
William E Copeland 220
Byron Creese 43
Lea K Davis 27
Eske M Derks 24
Enrico Domenici 221
Paul Elliott 84, 85, 197, 222
Andreas J Forstner 73, 76
Micha Gawlik 223
Joel Gelernter 19, 143, 224
Hans J Grabe 204-23-
Steven P Hamilton 225
Kristian Hveem 226, 227,228
Catherine John 169, 229
Jaakko Kaprio 141
Tilo Kircher 159
Marie-Odile Krebs 230
Karoline Kuchenbaecker 9,71
Mikael Landén 25, 127
Kelli Lehto 136
Douglas F Levinson 231
Qingqin S Li 232
Klaus Lieb 102
Yi Lu 25
Susanne Lucae 123
Jurjen J Luykx 15, 233
Patrik K Magnusson 25
Nicholas G Martin 24
Hilary C Martin 7
Andrew McQuillin 9
Christel M Middeldorp 62,234
Lili Milani 136
Ole Mors 30, 235

Daniel J Müller 101, 131,132, 236
Bertram Müller-Myhsok 38,237, 238
Albertine J Oldehinkel 115
Sara A Paciga 239
Colin NA Palmer 188
Peristera Paschou 196
Brenda WJH Penninx 45
Roy H Perlis 5, 6, 240
Giorgio Pistis 66
Renato Polimanti 18, 19
David J Porteous 64
Danielle Posthuma 241, 242
Ted Reichborn-Kjennerud40
Andreas Reif 31
Frances Rice 80, 243
Roland Ricken 3
Marcella Rietschel 2
Margarita Rivera 67, 244
Christian Rück 245
Catherine Schaefer 246
Srijan Sen 106, 247
Alessandro Serretti 105
Alkistis Skalkidou 137
Jordan W Smoller 5, 248,249
Frederike Stein 191
Murray B Stein 250, 251, 25
Patrick F Sullivan 25, 253
Martin Tesli 40
Thorgeir E Thorgeirsson 50
Henning Tiemeier 254, 255
Nicholas J Timpson 14
Rudolf Uher 256
Jens R Wendland 42
Thomas Werge 60, 179, 201,257, 258
Naomi R Wray 16, 259
Stephan Ripke 3, 248
Cathryn M Lewis 4, 260
Andrew M McIntosh 1, 261

PGC MDD Affiliations
1, Division of Psychiatry, University of Edinburgh, Edinburgh, UK
2, Department of Genetic Epidemiology in Psychiatry, Central Institute of Mental Health, Medical Faculty Mannheim, Heidelberg University, Mannheim, BW, DE
3, Department of Psychiatry and Psychotherapy, Charité – Universitätsmedizin Berlin, Berlin, BE, DE
4, Social, Genetic and Developmental Psychiatry Centre, King's College London, London, UK
5, Department of Psychiatry, Massachusetts General Hospital, Boston, MA, US
6, Department of Psychiatry, Harvard Medical School, Boston, MA, US
7, Human Genetics, Wellcome Sanger Institute, Hinxton, UK
8, NIHR Maudsley Biomedical Research Centre, King's College London, London, UK
9, Division of Psychiatry, University College London, London, UK
10, Department of Clinical Medicine, Aarhus University, Aarhus, DK
11, Department of Medical Biochemistry and Biophysics, Karolinska Institutet, Stockholm, SE
12, National Centre for Register-based Research, Aarhus University, Aarhus, DK
13, Department of Pediatric Neurology, Charité – Universitätsmedizin Berlin, Berlin, BE, DE
14, MRC Integrative Epidemiology Unit, University of Bristol, Bristol, UK
15, Department of Psychiatry and Neuropsychology, School for Mental Health and Neuroscience, Maastricht University Medical Centre, Maastricht, NL
16, Institute for Molecular Bioscience, University of Queensland, Brisbane, QLD, AU

17, Maurice Wohl Clinical Neuroscience Institute, Department of Basic and Clinical Neuroscience, King's College London, London, UK
18, Veterans Affairs Connecticut Healthcare System, West Haven, CT, US
19, Department of Psychiatry, Yale University School of Medicine, New Haven, CT, US
20, Department of Psychiatry, University of Munich, Munich, BY, DE
21, Institute of Psychiatric Phenomics and Genomics, University of Munich, Munich, BY, DE
22, Department of Psychiatry and Psychotherapy, University Hospital Bonn, Medical Faculty, University of Bonn, Bonn, DE
23, Institute of Human Genetics, University Hospital Bonn, Medical Faculty, University of Bonn, Bonn, DE
24, Mental Health and Neuroscience, QIMR Berghofer Medical Research Institute, Brisbane, QLD, AU
25, Department of Medical Epidemiology and Biostatistics, Karolinska Institutet, Stockholm, SE
26, Old Age Psychiatry, King's College London, London, UK
27, Department of Medicine, Division of Genetic Medicine, Vanderbilt University Medical Center, Nashville, TN, US
28, Department of Psychiatry and Psychotherapy, Fliedner Klinik Berlin, Berlin, BE, DE
29, Centre for Integrated Register-based Research, Aarhus University, Aarhus, DK
30, iPSYCH, The Lundbeck Foundation Initiative for Integrative Psychiatric Research, Aarhus, DK
31, Department of Psychiatry, Psychosomatic Medicine and Psychotherapy, Goethe University Frankfurt - University Hospital, Frankfurt am Main, DE
32, Discipline of Psychiatry, University of Adelaide, Adelaide, SA, AU
33, Department of Epidemiology, Columbia University Mailman School of Public Health, New York, NY, US
34, Department of Biomedicine and Centre for Integrative Sequencing, iSEQ, Aarhus University, Aarhus, DK
35, Center for Genomics and Personalized Medicine, Aarhus University, Aarhus, DK
36, Department of Clinical Neuroscience, Karolinska Institutet,, SE
37, Department of Neurology, Klinikum rechts der Isar, Technical University of Munich, Munich, BY,DE
38, Department of Translational Research in Psychiatry, Max Planck Institute of Psychiatry, Munich,BY, DE
39, Institute for Translational Psychiatry, University of Münster, Münster, NRW, DE-25-
40, Department of Mental Disorders, Norwegian Institute of Public Health, Oslo, NO
41, PROMENTA Research Center, Department of Psychology, University of Oslo, Oslo, NO
42, Research and Development, Takeda Pharmaceutical Company Limited, Cambridge, MA, US
43, Faculty of Health and Life Sciences, University of Exeter, Exeter, UK
44, Novo Nordisk Center for Protein Research, Department of Health Sciences, University of Copenhagen, Copenhagen, DK
45, Department of Psychiatry, Amsterdam Public Health and Amsterdam Neuroscience, Amsterdam UMC, Vrije Universiteit Amsterdam, Amsterdam, NL
46, Morphology and Genetics, Universidade Federal de Sao Paulo, Sao Paulo, SP, BR
47, Department of Psychiatry and Behavioral Sciences, Emory University School of Medicine, Atlanta, GA, US
48, Faculty of Nursing and Health Sciences, NORD University, Levanger, NO

49, Department of Mental Health, Faculty of Medicine and Health Sciences, Norwegian University of Science and Technology (NTNU), Trondheim, TRD, NO
50, deCODE Genetics / Amgen, Reykjavik, IS
51, K. G. Jebsen Center for Genetic Epidemiology, Department of Public Health and Nursing, Faculty of Medicine and Health Sciences, Norwegian University of Science and Technology (NTNU), Trondheim, TRD, NO
52, Department of Research and Innovation, Division of Clinical Neuroscience, Oslo University Hospital, Oslo, NO
53, Institute of Clinical Medicine, Faculty of Medicine, University of Oslo, Oslo, NO
54, Institute of Environmental Medicine, Unit of Integrative Epidemiology, Karolinska Institutet, Stockholm, SE
55, Department of Psychiatry and Psychotherapy, Charité – Universitätsmedizin Berlin, Berlin, DE
56, Department of Clinical Immunology, Roskilde University/Næstved Hospital, Roskilde, DK
57, Department of Clinical Immunology, Odense University Hospital, Odense, DK
58, Department of Psychiatry, Psychotherapy and Psychosomatics, Brandenburg Medical School Theodor Fontane, Neuruppin, BB, DE
59, Department of Psychiatry and Psychotherapy, Gender Research in Medicine, Institute of Sexology and Sexual Medicine, Charité – Universitätsmedizin Berlin, Berlin, BE, DE
60, iPSYCH, The Lundbeck Foundation Initiative for Integrative Psychiatric Research, Copenhagen, DK
61, Center for Neonatal Screening, Department for Congenital Disorders, Statens Serum Institut, Copenhagen, DK
62, Child Health Research Centre, University of Queensland, Brisbane, QLD, AU
63, Centre for Medical Informatics, Usher Institute, University of Edinburgh, Edinburgh, UK
64, Centre for Genomic & Experimental Medicine, Institute for Genetics and Cancer, University of Edinburgh, Edinburgh, UK
65, Department of Psychiatry and Mental Health, University of Cape Town, Cape Town, SA
66, Department of Psychiatry, Lausanne University Hospital and University of Lausanne, Prilly, VD, CH
67, Instituto de Investigación Biosanitaria ibs.GRANADA, Granada, ES
68, Department of Psychiatry, Faculty of Medicine and Institute of Neurosciences, Biomedical Research Centre (CIBM), University of Granada, Granada, ES
69, Université de Paris Cité, INSERM U1266, Institute of Psychiatry and Neuroscience of Paris, GHU Paris Psychiatry and Neuroscience, Paris, FR
70, Translational Biology, Biogen, Cambridge, MA, US
71, Nuffield Department of Population Health, University of Oxford, Oxford, UK
72, MRC Population Health Research Unit, University of Oxford, Oxford, UK
73, Institute of Neuroscience and Medicine (INM-1), Research Center Juelich, Juelich, DE
74, Human Genomics Research Group, Department of Biomedicine, University of Basel, Basel, CH
75, Institute of Medical Genetics and Pathology, University Hospital Basel, University of Basel, Basel,
CH-26-
76, Institute of Human Genetics, University of Bonn, School of Medicine & University Hospital Bonn, Bonn, DE
77, Nic Waals Institute, Lovisenberg Diakonale Hospital, Oslo, NO

78, Centre for Advanced Imaging, University of Queensland, Saint Lucia, QLD, AU
79, Psychological Medicine, Cardiff University, Cardiff, WLS, UK
80, Centre for Neuropsychiatric Genetics and Genomics, Cardiff University, Cardiff, WLS, UK
81, The Lothian Birth Cohorts, University of Edinburgh, Edinburgh, UK
82, Department of Biological Psychology & Amsterdam Public Health Research Institute, Vrije Universiteit Amsterdam, Amsterdam, NL
83, Department of Child and Adolescent Psychiatry, Psychosomatics and Psychotherapy, University Hospital Essen, Unversity of Duisburg-Essen, Duisburg, DE
84, MRC Centre for Environment and Health, School of Public Health, Imperial College London, London, UK
85, Imperial College Dementia Research Institute, Imperial College London, London, UK
86, Department of Psychiatry and Behavioral Sciences, Johns Hopkins University School of Medicine, Baltimore, MD, US
87, Department of Psychiatry and Psychotherapy, Research Group Stress Related Disorders, Central Institute of Mental Health, Medical Faculty Mannheim, Heidelberg University, Mannheim, BW, DE
88, Department of Clinical Immunology, Copenhagen University Hospital, Rigshospitalet, Copenhagen, CPH, DK
89, Department of Clinical Immunology, Aarhus University Hospital, Aarhus, DK
90, Department of Psychiatry, Istanbul University, Istanbul, TR
91, Department of Medical Genetics, Oslo University Hospital, Oslo, OSL, NO
92, NORMENT, Department of Clinical Science, University of Bergen, Bergen, NO
93, Virginia Institute for Psychiatric & Behavioral Genetics, Virginia Commonwealth University, Richmond, VA, US
94, Psychiatry Department / Huntsman Mental Health Institute, University of Utah School of Medicine, Salt Lake City, UT, US
95, Center for Genomic Research, University of Utah School of Medicine, Salt Lake City, UT, US
96, Department of Psychiatry and Psychotherapy, Medical Center, University of Freiburg, Faculty of Medicine, University of Freiburg, Freiburg,DE
97, Division of Mental Health Care, St. Olavs Hospital, Trondheim University Hospital, Trondheim, TRD,NO
98, Department of Psychiatry, Sørlandet Hospital, Kristiansand, AG, NO
99, University of Oslo, NORMENT Centre, Institute of Clinical Medicine, Oslo, OSL, NO
100, Center for Genomic Medicine, Massachusetts General Hospital, Boston, MA, US
101, Centre for Addiction and Mental Health, Toronto, ON, CA
102, Department of Psychiatry and Psychotherapy, University Medical Center of the Johannes Gutenberg University Mainz, Mainz, DE
103, Mental Health Center Copenhagen, Mental Health Services Capital Region of Denmark, Copenhagen, DK
104, Faculty of Health Science, Department of Clinical Medicine, University of Copenhagen, Copenhagen, DK
105, Department of Biomedical and Neuromotor Sciences, University of Bologna, Bologna, IT
106, Michigan Neuroscience Institute, University of Michigan, Ann Arbor, MI, US
107, Wolfson Institute of Population Health, Queen Mary University of London, London, UK

108, School of Computing and Mathematical Sciences, University of Leicester, Leicester, UK
109, Department of Epidemiology and Biostatistics, Imperial College London, London, UK
110, Discipline of Psychiatry, School of Medicine, Trinity College Dublin, Dublin, IE
111, Bioinformatics Research Centre, Aarhus University, Aarhus, DK
112, School of Engineering, University of Iceland, Reykjavik, IS-27-
113, Danish Headache Centre, Department of Neurology, Rigshospitalet, Glostrup, DK
114, Faculty of Medicine, Department of Psychiatry, University of Iceland, Reykjavik, IS
115, Department of Psychiatry, University of Groningen, University Medical Center Groningen, Groningen, NL
116, MRC Human Genetics Unit, Institute for Genetics and Cancer, University of Edinburgh, Edinburgh, UK
117, Brain and Mind Centre, University of Sydney, Sydney, NSW, AU
118, Department of Epidemiology Research, Statens Serum Institut, Copenhagen, DK
119, Interfaculty Institute for Genetics and Functional Genomics, Department of Functional Genomics, University Medicine Greifswald, Greifswald, MV, DE
120, Roche Pharmaceutical Research and Early Development, Pharmaceutical Sciences, Roche Innovation Center Basel, F. Hoffmann-La Roche Ltd, Basel, CH
121, SleepWell Research Program and Department of Psychology and Logopedics, University of Helsinki, Helsinki, FI
122, Blizard Institute, Barts and the London School of Medicine and Dentistry, Queen Mary University of London, London, UK
123, Max Planck Institute of Psychiatry, Munich, BY, DE
124, Department of Psychiatry, University of Helsinki, Helsinki, FI
125, 23andMe Research Team, 23andMe, Inc., Sunnyvale, CA, US
126, Department of Psychological Medicine, University of Worcester, Worcester, UK
127, Institution of Neuroscience and Physiology, University of Gothenburg, Gothenburg, SE
128, Department of Psychiatry and Psychotherapy, Medical University of Vienna, Vienna, AT
129, Department of Psychiatry, Virginia Commonwealth University, Richmond, VA, US
130, Health Care Policy, Harvard Medical School, Boston, MA, US
131, Department of Psychiatry, University of Toronto, Toronto, ON, CA
132, Department of Pharmacology & Toxicology, University of Toronto, Toronto, ON, CA
133, Department of Genetics, Rutgers University, Piscataway, NJ, US
134, Department of Psychiatry, Perelman School of Medicine, University of Pennsylvania, Philadelphia, PA, US
135, Mental Illness Research, Education and Clinical Center, Crescenz VA Medical Center, Philadelphia, PA, US
136, Estonian Genome Centre, Institute of Genomics, University of Tartu, Tartu, EE
137, Department of Women's and Children's Health, Uppsala University, Uppsala, SE
138, Department of Epidemiology and Health Systems, Center for Primary Care and Public Health, Lausanne, VD, CH
139, Swiss Institute of Bioinformatics, Lausanne, VD, CH

140, Department of Computational Biology, University of Lausanne, Lausanne, VD, CH
141, Institute for Molecular Medicine Finland - FIMM, University of Helsinki, Helsinki, FI
142, Department of Psychiatry, Washington University School of Medicine in St. Louis, St. Louis, MO, US
143, Psychiatry, Veterans Affairs Connecticut Healthcare System, West Haven, CT, US
144, Department of Psychiatry, Yale University, New Haven, CT, US
145, Department of Epidemiology and Biostatistics, School of Public Health, Peking University, Beijing, CN
146, Peking University Center for Public Health and Epidemic Preparedness & Response, Peking University, Beijing, CN
147, Mental Health, NHS 24, Glasgow, UK
148, Royal Edinburgh Hospital, NHS Lothian, Edinburgh, UK
149, Department of Human and Molecular Genetics, Virginia Commonwealth University, Richmond, VA, USA-28-
150, Virginia Institute for Psychiatric and Behavioral Genetics, Virginia Commonwealth University, Richmond, VA, USA
151, Department of Psychiatry and Psychotherapy, University of Bonn, Bonn, DE
152, Center for Translational and Computational Neuroimmunology, Columbia University Medical Center, New York, NY, US
153, Institute for Translational Neuroscience, University of Münster, Münster, NRW, DE
154, Novo Nordisk Foundation Center for Basic Metabolic Research, Faculty of Health Science, Copenhagen University, Copenhagen, DK
155, Department of Nursing, Faculty of Health Sciences and Institute of Neurosciences, Biomedical Research Centre (CIBM), University of Granada, Granada, ES
156, School of Public Health, University of Queensland, Brisbane, QLD, AU
157, DZHK (German Centre for Cardiovascular Research), Partner Site Greifswald, Greifswald, MV, DE
158, Institute of Clinical Chemistry and Laboratory Medicine, University Medicine Greifswald, Greifswald, MV, DE
159, Department of Psychiatry, University of Marburg, Marburg, DE
160, Department of Clinical Immunology, Aalborg University Hospital, Aalborg, DK
161, Department of Epidemiology, University of Groningen, University Medical Center Groningen, Groningen, NL
162, Department of Health, Science and Technology, Aalborg University, Aalborg, DK
163, Centre for Integrative Sequencing, iSEQ, Aarhus University, Aarhus, DK
164, Department of Biomedicine-Human Genetics, Aarhus University, Aarhus, DK
165, Population Health, QIMR Berghofer Medical Research Institute, Brisbane, QLD, AU
166, The Fraser Institute, Faculty of Medicine, University of Queensland, Brisbane, QLD, AU
167, Humus, Reykjavik, IS
168, Department of Clinical Medicine, University of Copenhagen, Copenhagen, CPH, DK
169, Department of Population Health Sciences, University of Leicester, Leicester, UK
170, Department of Psychiatry, Universidade Federal de Sao Paulo, Sao Paulo, SP, BR
171, Department of Psychiatry, Rutgers University, Piscataway, NJ, US
172, Department of Clinical Immunology, Zealand University Hospital, Køge, DK

173, Department of Psychiatry and Behavioral Sciences, SUNY Downstate Health Sciences University, Brooklyn, NY, US
174, NMD Pharma, Lexington, MA, US
175, Psychiatry, University of Pittsburgh Medical Centre, Pittsburgh, PA, US
176, Psychiatry, Universidade Federal do Rio Grande do Sul, Porto Alegre, BR
177, Department of Psychiatry, University Medical Center Groningen, Groningen, NL
178, Research School of Behavioural and Cognitive Neurosciences (BCN), University of Groningen, Groningen, NL
179, Institute of Biological Psychiatry, Mental Health Center Sct. Hans, Mental Health Services Capital Region of Denmark, Copenhagen, DK
180, Neurogenomics Division, The Translational Genomics Research Institute (TGEN), Phoenix, AZ, US
181, Human Genetics Branch, NIMH Division of Intramural Research Programs, Bethesda, MD, US
182, Department of Psychiatry and Psychotherapy, University Medical Center Göttingen, Goettingen, NI, DE
183, Division of Cancer Epidemiology and Genetics, National Cancer Institute, Bethesda, MD, US
184, School of Medicine and Dentistry, James Cook University, Townsville, QLD, AU
185, Division of Mental Health and Addiction, Oslo University Hospital, Oslo, OSL, NO
186, NORMENT, Institute of Clinical Medicine, University of Oslo, Oslo, OSL, NO
187, Institute of Health and Wellbeing, University of Glasgow, Glasgow, UK
188, Division of Population Health and Genomics, Ninewells Hospital and School of Medicine, University of Dundee, Dundee, UK
189, Faculty of Medicine, University of Iceland, Reykjavik, IS-29-
190, SAMRC Unit on Risk & Resilience in Mental Disorders, Department of Psychiatry and Mental Health, University of Cape Town, Cape Town,SA
191, Department of Psychiatry and Psychotherapy, University of Marburg, Marburg, HE, DE
192, Department of Psychiatry, Psychotherapy and Psychosomatics, Dr. Fontheim Mentale Gesundheit, Liebenburg, DE
193, Institute of Epidemiology and Social Medicine, University of Münster, Münster, NRW, DE
194, Institute for Community Medicine, University Medicine Greifswald, Greifswald, MV, DE
195, Wolfson Centre for Young People's Mental Health, Division of Psychological Medicine and Clinical Neurosciences, Cardiff University, Cardiff, WLS, UK
196, Department of Biological Sciences, Purdue University, West Lafayette, IN, US
197, Imperial College BHF Centre for Research Excellence, Imperial College London, London, UK
198, Genomics Program, University of South Florida College of Public Health, Tampa, FL, US
199, Department of Internal Medicine, Erasmus University Medical Center Rotterdam, Rotterdam, NL
200, Management Section, Statens Serum Institut, Copenhagen, DK
201, Department of Clinical Medicine, University of Copenhagen, Copenhagen, DK
202, Xperimed LLC, Basel, CH
203, Psychiatry, USUHS, Bethesda, US
204, Department of Psychiatry and Psychotherapy, University Medicine Greifswald, Greifswald, MV, DE

205, Department of Psychiatry, Leiden University Medical Center, Leiden, NL
206, Department of Epidemiology and Population Health, Albert Einstein College of Medicine, Bronx, NY, US
207, Department of Psychiatry, Columbia University College of Physicians and Surgeons, New York, NY, US
208, Division of Epidemiology, New York State Psychiatric Institute, New York, NY, US
209, Department of Neurology, Oslo University Hospital, Oslo, NO
210, HUNT All-In Psychiatry
211, China Kadoorie Biobank Collaborative Group
212, Genes & Health Research Team
213, KG Jebsen Centre for Neurodevelopmental Research, University of Oslo, Oslo, OSL, NO
214, Department of Psychiatry, University of Melbourne, Melbourne, VIC, AU
215, Florey Institute of Neuroscience and Mental Health, University of Melbourne, Melbourne, VIC, AU
216, Department of Psychiatry, University of Münster, Münster, NRW, DE
217, Computational Health Centre, Helmholtz Zentrum München, Neuherberg, DE
218, School of Medicine, Technical University of Munich, Munich, BY, DE
219, Helmholtz Pioneer Campus, Helmholtz Zentrum München, Neuherberg, DE
220, Department of Psychiatry, University of Vermont, Burlington, VT, US
221, Department of Cellular, Computational and Integrative Biology, Università degli Studi di Trento, Trento, IT
222, Imperial College Biomedical Research Centre, Imperial College London, London, UK
223, Department of Psychiatry, Psychosomatics and Psychotherapy, Julius-Maximilians-Universität Würzburg, Würzburg, DE
224, Department of Genetics, Department of Neuroscience, Yale University School of Medicine, New Haven, CT, US
225, Psychiatry, Kaiser Permanente Northern California, San Francisco, CA, US
226, K. G. Jebsen Center for Genetic Epidemiology, Department of Public Health and Nursing, Faculty of Medicine and Health Sciences, Norwegian University of Science and Technology (NTNU), Trondheim, NO
227, HUNT Research Center, Department of Public Health and Nursing, Faculty of Medicine and Health Sciences, Norwegian University of Science and Technology (NTNU), Trondheim, NO-30-
228, Department of Research, Innovation and Education, St. Olavs Hospital, Trondheim University Hospital, Trondheim, NO
229, NIHR Leicester Biomedical Research Centre, Glenfield Hospital, Leicester, UK
230, Pathophysiology of Psychiatric Diseases, INSERM, Univ Paris Cité, GHU Paris, Paris, FR
231, Department of Psychiatry & Behavioral Sciences, Stanford University, Stanford, CA, US
232, Neuroscience Therapeutic Area, Janssen Research and Development, LLC, Titusville, NJ, US
233, Second Opinion Outpatient Clinic, GGNet Mental Health, Warnsveld, NL
234, Child and Youth Mental Health Service, Children's Health Queensland Hospital and Health Service, Brisbane, QLD, AU
235, Psychosis Research Unit, Aarhus University Hospital-Psychiatry, Aarhus, DK
236, Department of Psychiatry, Psychosomatics and Psychotherapy, University Hospital of Würzburg, Würzburg, DE

237, Munich Cluster for Systems Neurology (SyNergy), Munich, BY, DE
238, University of Liverpool, Liverpool, UK
239, Human Genetics and Computational Biomedicine, Pfizer Global Research and Development, Groton, CT, US
240, Centre for Quantitative Health, Massachusetts General Hospital, Boston, MA, US
241, Child and Adolescent Psychiatry, Amsterdam UMC, Vrije Universiteit Amsterdam, Amsterdam, NL
242, Complex Trait Genetics, Vrije Universiteit Amsterdam, Amsterdam, NL
243, Wolfson Centre for Young People's Mental Health, Division of Psychological Medicine and Clinical Neurosciences, Cardiff University, Cardiff, UK
244, Department of Biochemistry and Molecular Biology II, Faculty of Pharmacy and Institute of Neurosciences, Biomedical Research Centre (CIBM), University of Granada, Granada, ES
245, Department of Clinical Neuroscience, Karolinska Institutet, Stockholm, SE
246, Division of Research, Kaiser Permanente Northern California, Oakland, CA, US
247, Department of Psychiatry, University of Michigan, Ann Arbor, MI, US
248, Stanley Center for Psychiatric Research, Broad Institute of MIT and Harvard, Cambridge, MA, US
249, Psychiatric and Neurodevelopmental Genetics Unit, Massachusetts General Hospital, Boston, MA, US
250, Psychiatry, UCSD School of Medicine, La Jolla, CA, US
251, Public Health, UCSD School of Public Health, La Jolla, CA, US
252, Psychiatry, Veterans Affairs San Diego Healthcare System, San Diego, CA, US
253, Departments of Genetics and Psychiatry, University of North Carolina at Chapel Hill, Chapel Hill, NC, US
254, Child and Adolescent Psychiatry, Erasmus University Medical Center Rotterdam, Rotterdam, NL
255, Social and Behavioral Science, Harvard T.H. Chan School of Public Health, Boston, MA, US
256, Psychiatry, Dalhousie University, Halifax, NS, CA
257, Institute of Biological Psychiatry, Mental Health Center Sct. Hans, Copenhagen University Hospital, Mental Health Services, Copenhagen, DK
258, GLOBE Institute, Lundbeck Foundation Centre for Geogenetics, University of Copenhagen, Copenhagen, DK
259, Queensland Brain Institute, University of Queensland, Brisbane, QLD, AU
260, Department of Medical & Molecular Genetics, King's College London, London, UK
261, Institute for Genomics and Cancer, University of Edinburgh, Edinburgh, UK-31-S1: Previous MR evidence for micronutrients in major depression

References

1. Michaelsson K, Melhus H, Larsson SC. Serum 25-Hydroxyvitamin D Concentrations and Major Depression: A Mendelian Randomization Study. Nutrients. 2018;10(12).
2. Milaneschi Y, Peyrot WJ, Nivard MG, Mbarek H, Boomsma DI, Penninx BWJH. A role for vitamin D and omega-3 fatty acids in major depression? An exploration using genomics. Transl Psychiat. 2019;9.
3. Mulugeta A, Lumsden A, Hypponen E. Relationship between Serum 25(OH)D and Depression: Causal Evidence from a Bi-Directional Mendelian Randomization Study. Nutrients. 2020;13(1).
4. Libuda L, Laabs BH, Ludwig C, Buhlmeier J, Antel J, Hinney A, et al. Vitamin D and the Risk of Depression: A Causal Relationship? Findings from a Mendelian Randomization Study. Nutrients. 2019;11(5).
5. Revez JA, Lin T, Qiao Z, Xue A, Holtz Y, Zhu Z, et al. Genome-wide association study identifies 143 loci associated with 25 hydroxyvitamin D concentration. Nat Commun. 2020;11(1):1647.
6. Mollehave LT, Skaaby T, Simonsen KS, Thuesen BH, Mortensen EL, Sandholt CH, et al. Association studies of genetic scores of serum vitamin B12 and folate levels with symptoms of depression and anxiety in two danish population studies. Eur J Clin Nutr. 2017;71(9):1054-60.
7. Cheng WW, Zhu Q, Zhang HY. Mineral Nutrition and the Risk of Chronic Diseases: A Mendelian Randomization Study. Nutrients. 2019;11(2):12.
8. Howard DM, Adams MJ, Clarke TK, Hafferty JD, Gibson J, Shirali M, et al. Genome-wide meta-analysis of depression identifies 102 independent variants and highlights the importance of the prefrontal brain regions. Nat Neurosci. 2019;22(3):343-52.
9. Hyde CL, Nagle MW, Tian C, Chen X, Paciga SA, Wendland JR, et al. Identification of 15 genetic loci associated with risk of major depression in individuals of European descent. Nat Genet. 2016;48(9):1031-6.
10. Wray NR, Ripke S, Mattheisen M, Trzaskowski M, Byrne EM, Abdellaoui A, et al. Genome-wide association analyses identify 44 risk variants and refine the genetic architecture of major depression. Nat Genet. 2018;50(5):668-81.
11. Tanaka T, Scheet P, Giusti B, Bandinelli S, Piras MG, Usala G, et al. Genome-wide association study of vitamin B6, vitamin B12, folate, and homocysteine blood concentrations. Am J Hum Genet. 2009;84(4):477-82.
12. Grarup N, Sulem P, Sandholt CH, Thorleifsson G, Ahluwalia TS, Steinthorsdottir V, et al. Genetic architecture of vitamin B12 and folate levels uncovered applying deeply sequenced large datasets. Plos Genet. 2013;9(6):e1003530.
13. van Meurs JBJ, Pare G, Schwartz SM, Hazra A, Tanaka T, Vermeulen SH, et al. Common genetic loci influencing plasma homocysteine concentrations and their effect on risk of coronary artery disease. Am J Clin Nutr. 2013;98(3):668-76.
14. Mondul AM, Yu K, Wheeler W, Zhang H, Weinstein SJ, Major JM, et al. Genome-wide association study of circulating retinol levels. Hum Mol Genet. 2011;20(23):4724-31.
15. Ferrucci L, Perry JR, Matteini A, Perola M, Tanaka T, Silander K, et al. Common variation in the beta-carotene 15,15'-monooxygenase 1 gene affects circulating levels of carotenoids: a genome-wide association study. Am J Hum Genet. 2009;84(2):123-33.

16. Jiang X, O'Reilly PF, Aschard H, Hsu YH, Richards JB, Dupuis J, et al. Genome-wide association study in 79,366 European-ancestry individuals informs the genetic architecture of 25-hydroxyvitamin D levels. Nat Commun. 2018;9(1):260.
17. O'Seaghdha CM, Wu H, Yang Q, Kapur K, Guessous I, Zuber AM, et al. Meta-analysis of genome-wide association studies identifies six new Loci for serum calcium concentrations. Plos Genet. 2013;9(9):e1003796.
18. Meyer TE, Verwoert GC, Hwang SJ, Glazer NL, Smith AV, van Rooij FJA, et al. Genome-Wide Association Studies of Serum Magnesium, Potassium, and Sodium Concentrations Identify Six Loci Influencing Serum Magnesium Levels. Plos Genet. 2010;6(8).
19. Kestenbaum B, Glazer NL, Kottgen A, Felix JF, Hwang SJ, Liu YM, et al. Common Genetic Variants Associate with Serum Phosphorus Concentration. J Am Soc Nephrol. 2010;21(7):1223-32.
20. Benyamin B, Esko T, Ried JS, Radhakrishnan A, Vermeulen SH, Traglia M, et al. Novel loci affecting iron homeostasis and their effects in individuals at risk for hemochromatosis. Nature Communications. 2014;5.
21. Evans DM, Zhu G, Dy V, Heath AC, Madden PAF, Kemp JP, et al. Genome-wide association study identifies loci affecting blood copper, selenium and zinc. Human Molecular Genetics. 2013;22(19):3998-4006.
22. Ng E, Lind PM, Lindgren C, Ingelsson E, Mahajan A, Morris A, et al. Genome-wide association study of toxic metals and trace elements reveals novel associations. Hum Mol Genet. 2015;24(16):4739-45.
23. Arakawa K, Kono N, Yamada Y, Mori H, Tomita M. KEGG-based pathway visualization tool for complex omics data. In Silico Biol. 2005;5(4):419-23.
24. Patel A, Ye T, Xue H, Lin Z, Xu S, Woolf B, et al. MendelianRandomization v0.9.0: updates to an R package for performing Mendelian randomization analyses using summarized data. Wellcome Open Res. 2023;8:449.
